# Comparison of homologous and heterologous vaccination strategies for combating disease caused by *Burkholderia pseudomallei*

**DOI:** 10.3389/fimmu.2025.1596265

**Published:** 2025-06-20

**Authors:** Sergei S. Biryukov, Christopher K. Cote, Christopher P. Klimko, Jennifer L. Dankmeyer, Nathaniel O. Rill, Melissa Hunter, Michael L. Davies, Zander M. Hedrick, Jennifer L. Shoe, Lindsey K. Schmidt, Caitlyn E. Orne, Ju Qiu, Susan L. Welkos, Mary N. Burtnick, Paul J. Brett, David DeShazer

**Affiliations:** ^1^ United States Army Medical Research Institute of Infectious Diseases (USAMRIID), Bacteriology Division, Frederick, MD, United States; ^2^ Department of Microbiology and Immunology, University of Nevada, Reno School of Medicine, Reno, NV, United States; ^3^ United States Army Medical Research Institute of Infectious Diseases (USAMRIID), Biostatistics Division, Frederick, MD, United States; ^4^ Department of Microbiology and Immunology, Faculty of Tropical Medicine, Mahidol University, Bangkok, Thailand

**Keywords:** melioidosis, *Burkholderia pseudomallei*, vaccines, heterologous, mice, protection, immunity

## Abstract

**Introduction:**

Melioidosis is a major cause of disease and mortality in endemic tropical regions, and the etiologic agent, *Burkholderia pseudomallei*, is being isolated increasingly from an expanded range of environmental and clinical sources in locations including the United States. The disease can have multi-faceted clinical presentations and requires a complex and protracted treatment regimen which is confounded by resistance of this microbe to numerous antibiotics. Thus, prophylactic countermeasures are needed; however, a vaccine has yet to be licensed for human use. Since *B. pseudomallei* is classified as a Tier 1 select agent, the development of a safe and effective vaccine is both a military and public health need. Our laboratories have focused on the development of vaccines composed of live attenuated strains and defined subunit antigens.

**Methods:**

In the current study, we evaluated homologous and heterologous combinations of candidate subunits and live vaccines in a murine aerosol model of melioidosis to determine the effects of vaccine composition and delivery scheme on protection in conjunction with immune responses and bacterial clearance.

**Results:**

Both strategies provided significant protection against lethal aerosol challenges, and the accumulated data support that a heterologous vaccination strategy employing capsular polysaccharide conjugate and Hcp1 subunits and a live but highly safe capsular polysaccharide-producing surrogate strain of *B. thailandensis* is an effective and potentially agile prophylactic strategy.

## 1 Introduction

*Burkholderia pseudomallei*, a gram-negative bacillus found in various environmental settings, is a potential biothreat agent and the causative agent of melioidosis, a disease of significant public health importance (1–6). Infections with *B. pseudomallei* are commonly acquired through inhalation or direct cutaneous inoculation of the organism into the human or animal host (2–4). Melioidosis is a multi-faceted disease with manifestations ranging from acute and rapidly fatal to protracted and chronic. The disease is acquired most often by individuals with co-existing risk factors including diabetes, excessive alcohol use, chronic lung or renal disease, and other immunocompromising conditions (2–5, 7, 8). Melioidosis usually presents as an acute illness (pneumonia, bacteremia, or localized infection), but patients can have chronic infections, with symptoms lasting many months, or can be sub-clinically infected (2, 8–10). The treatment regimen required to resolve *B. pseudomallei* infections is multi-phased and lengthy, and can be complicated since the bacterium is resistant to multiple antibiotics and has a complex *in vivo* lifecycle that involves biofilm formation, facultative intracellular existence, and treatment-related phenotypic changes (2–4, 11–15).

Melioidosis is a major cause of disease and mortality in endemic subtropical and tropical regions, especially Southeast Asia and northern Australia (2–5, 7). Recent improvements in environmental isolation of *B. pseudomallei* and the increased identification of melioidosis cases in locations such as India, Africa, and the Americas make the case for recognizing a wider ecological range and greater significance of *B. pseudomallei* (2–5, 7, 16, 17). Such studies have revealed that the incidence and extent of the disease are likely highly underreported (5, 6). This situation is attributed to the fact that melioidosis is often misdiagnosed due to its nonspecific and variable symptomatology and its clinical resemblance to other infections, such as tuberculosis. Difficulties in laboratory isolation and identification of the agent have also contributed to lower numbers of confirmed cases being reported (2, 4, 18–21).

*B. pseudomallei* and the closely related saprophytic species *Burkholderia thailandensis* have recently been isolated from the soil and water in areas once considered to be inhospitable to these microbes, a finding attributed in part to climate change (6, 16, 22–25). Importantly, melioidosis is being diagnosed more often in the United States. While most of the cases have been related to either traveling to areas with endemic melioidosis or to exposure to contaminated imported products or pets (22, 26–28), three locally-acquired cases in Mississippi support that *B. pseudomallei* is endemic in the southern United States (22, 29). Similar cases in Texas were suggestive of local acquisition even though attempts to isolate comparable environmental strains were not successful (25). Importantly, the closely related opportunistic pathogen *B. thailandensis* was isolated from water in Texas and Puerto Rico and from soil in Mississippi, further supporting the likelihood of endemicity of these *Burkholderia* species (23).

The complexities of disease diagnosis and treatment, wide geographic distribution of the agent, and its potential for adversarial use necessitate the development of effective prophylactic countermeasures. Our laboratories have focused on the development and *in vivo* evaluation of the protective efficacy of vaccines composed of live attenuated and defined subunit antigens (30–36). The composition of these vaccines is detailed in recent reports (31, 36) and in the present study. The goal of this research was to compare homologous and heterologous combinations of subunit and live attenuated vaccine candidates in a mouse model of inhalational melioidosis to determine the effects of vaccine composition and delivery schemes with respect to protection, immune responses, and bacterial clearance.

## 2 Materials and methods

### 2.1 Preparation of vaccine candidates and bacteria

The live attenuated vaccine (LAV) was composed of *B. thailandensis* E555, a strain that produces a *B. pseudomallei*-like 6-deoxyheptan capsular polysaccharide (CPS) and harbors a deletion mutation in *ilvI* that renders it auxotrophic for isoleucine, leucine, and valine (E555 Δ*ilvI*); the LAV was constructed at the USAMRIID. The subunit vaccines were prepared at the University of Nevada, Reno as detailed previously (32–35). The subunit vaccines contained *B. thailandensis* E555 6-deoxyheptan capsular polysaccharide CPS conjugated to the carrier protein Cross-Reactive Material 197 (CRM197, genetically detoxified version of diphtheria toxin) to form CPS-CRM197, alone or combined with recombinant *B. pseudomallei* hemolysin co-regulated protein 1 (Hcp1). These antigens were formulated in sterile PBS (ThermoFisher Scientific, Waltham, MA), 250 µg Alhydrogel adjuvant (Brenntag Biosector, Denmark) with or without 10 µg of the immunostimulatory oligodeoxynucleotide (ODN) CpG 2006 (ODN 7909) (InvivoGen, San Diego, CA) as indicated. For each individual study, the composition of all LAV and/or subunit vaccine candidates used is outlined in the figures and legends.

The LAV strain E555 Δ*ilvI* was constructed, cultured, and prepared as described previously (30, 37). The aerosol challenge strain, *B. pseudomallei* K96243, is a virulent strain from Thailand that is often used in laboratory studies (38–40) and was grown and prepared as described previously (30, 31, 41). In brief, a frozen stock of *B. pseudomallei* K96243 was grown in tryptose broth with 4% glycerol and 5% NaCl (GTB) at 37°C with shaking until late log phase (approximately 16 h). The bacteria were harvested, resuspended in GTB, and quantified by OD_620_ estimation. The actual delivered dose of bacteria, as the number of colony forming units (CFU), was verified by plate counts on sheep blood agar plates.

### 2.2 Animals and vaccination conditions

All animal research was conducted under an animal use protocol approved by the USAMRIID Institutional Animal Care and Use Committee (IACUC) in compliance with the Animal Welfare Act, Public Health Service Policy, and other federal statutes and regulations relating to animals and experiments involving animals. The facility where this research was conducted is accredited by the AAALAC International and adheres to the principles stated in *The Guide for the Care and Use of Laboratory Animals* (National Research Council, 2011).

Female C57BL/6 mice were obtained from Charles River (Frederick, MD) and were 7–10 weeks of age at time of vaccination. All vaccines were injected subcutaneously (sc) on days 0 (prime) and 28 (boost) in a total volume of 200 µl. The subunit vaccines were divided, with 100 µl injected in each hind flank, as described previously (31). The E555 Δ*ilvI* LAV was delivered at target doses of ~ 10^7^ CFU. Control mice were inoculated with PBS alone or with the Alhydrogel and CpG as indicated.

### 2.3 Exposure to the aerosolized challenge strain

Vaccinated and unvaccinated control groups of C57BL/6 mice were challenged with *B. pseudomallei* K96243, prepared as detailed above, by a whole-body aerosol route approximately one month after the last vaccine dose. For the exposures, mice were transferred to wire mesh cages and were placed in a whole-body aerosol chamber within a class three biological safety cabinet located inside a BSL-3 laboratory. Mice were then exposed to aerosols of *B. pseudomallei* suspensions created by a three-jet Collison nebulizer (41). Samples were collected from the all-glass impinger (AGI) vessel and analyzed bacteriologically to determine the inhaled dose of *B. pseudomallei* in CFU, as described above.

### 2.4 Clinical observations and sample collections

Challenged mice were observed at least daily for 60 days for clinical signs of illness, as described previously (41). Early intervention endpoints were used during all studies and mice were euthanized when moribund, according to an endpoint score sheet. Animals were scored on a scale of 0–9: 0–2 = no significant clinical signs (e.g., slightly ruffled fur); 3–4 = significant clinical symptoms such as subdued behavior, hunched appearance, absence of grooming, hind limb issues of varying severity and/or pyogranulomatous swelling of varying severity (increased monitoring was warranted); > 5 = distress. Those animals receiving a score of > 5 were euthanized with a pentobarbital-based euthanasia solution given intraperitoneally (IP). Animals which survived were euthanized at the study endpoint and necropsied for tissue collection for bacteriological analyses. For sample collections, mice were deeply anesthetized with approximately 0.3 ml/20 g of body weight with a mixture of ketamine (10 mg/ml)-acepromazine (1 mg/ml)-xylazine (2 mg/ml), underwent a terminal blood collection via the axillary vessels, and then were euthanized by cervical dislocation prior to organ harvesting. When mice met pre-determined euthanasia criteria and they were not in the sampling cohorts, mice were euthanized by CO_2_ exposure or by barbiturate overdose through intraperitoneal injection (approximately 0.15 ml for 20 g of body weight) of Euthasol^®^ euthanasia solution (or equivalent) and then death was confirmed by cervical dislocation.

### 2.5 Bacteriology

The number of viable bacteria present in tissues of mice were determined on day 3 post challenge and for survivors at the study endpoint. The tissues collected from necropsied mice included lung, spleen, and blood. Lung and spleen tissues were weighed, rinsed in sterile PBS, suspended in 1 ml of PBS, and homogenized with disposable PRECISION™ homogenizers (Covidien, Dublin, Ireland). The CFU of the homogenates were determined on sheep blood agar plates. Undiluted homogenate and 10-fold dilutions in PBS were plated in duplicate to determine sterility. The values reported were the geometric mean (GM) and geometric standard deviation (GSD) of CFU/ml of blood and CFU/g of organ. The limit of detection (LOD) was approximately 50 CFU/ml blood or 5 CFU/organ.

### 2.6 ELISAs

The total immunoglobulin G (IgG) responses to the vaccine antigens were assessed using blood collected one to six days before challenge in immunized mice. The blood was obtained via submandibular collection techniques from 15 mice from each group and combined in pools of three mice each for a total of five samples per vaccine candidate. IgG titers were determined by ELISA as described previously (41), using some or all of the following capture antigens: irradiated whole cells of *B. pseudomallei* K96243 (BpK), purified CPS, and Hcp1. The titer results were reported as the GM and geometric standard error (GSE) of the reciprocal of the highest dilution giving a mean OD of at least 0.100 ± 1 SD at 450 nm with a reference filter (570 nm). The limit of detection was a reciprocal titer of 50 and samples with an antibody titer of ≤ 50 were considered negative. In some ELISAs, IgG1 and IgG2c titers were also determined, as described previously (42). The secondary antibodies used in the ELISAs were goat anti-mouse IgG (or IgG subclass) horseradish peroxidase conjugates obtained from Southern Biotechnology Associates, Inc. (Birmingham, AL).

### 2.7 Splenocyte and organ homogenate collection and preparation

Prior to challenge, spleens were collected from necropsied C57BL/6 mice, and the splenocytes were isolated and prepared for *in vitro* analysis as described previously (30, 41, 43). In brief, splenocytes were extracted through manual disruption of spleens in RPMI 1640 (ThermoFisher, Grand Island, NY). The large debris was removed, the splenocyte-containing supernatant was collected and washed in RPMI 1640, and the cell pellet was resuspended in Ammonium-Chloride-Potassium (ACK) Lysis buffer (Lonza, Walkersville, MD). After incubation for 5 min at room temperature, RPMI 1640 was added to stop the reaction. After removal of cell debris and washing of the cells, the final splenocyte pellet was resuspended in CTL-Test Medium (CTL, Shaker Heights, OH) with 1% L-Glutamine. The cells were counted with a TC20 Cell Counter (BioRad) and splenocytes were diluted to a concentration of 10 x 10^6^/ml in RPMI complete medium and 8 x 10^6^/ml in CTL-Medium for use in the Luminex and ELISpot *in vitro* stimulation assays, respectively. Recall antigen Hcp1 was used in the re-stimulation assays at 5 or 10 µg/ml, as indicated.

Following challenges, spleen, and lung homogenates were processed as described in the Bacteriology section, above. Aliquots of the homogenates were frozen and stored at -80°C until analysis of cytokines. Prior to analysis, frozen samples were exposed to approximately 21 kGy γ-radiation, sterility was verified, and they were refrozen for storage. When ready for use, the samples were thawed, centrifuged at 10,000 x g for 10 min, and the supernatants were then examined for cytokine expression.

### 2.8 ELISpot assays

Cellular immune responses in the immunized animals were assessed using Enzyme-Linked ImmunoSpot (ELISpot) assays that measured IFN-γ secretion by splenocytes (predominantly activated T cells). The splenocytes were incubated with Hcp1 to stimulate the cells and the assays conducted as described previously (44). Cells from each mouse were evaluated in duplicate, in independent stimulation conditions. A solution of phorbol 12-myristate 13-acetate (PMA; 100 ng/ml) and ionomycin (0.5 µg/ml) was used as the positive control stimulant and resulted in uniformly strong signals, while medium alone was used as a negative control (data not shown). Briefly, 96-well plates were coated overnight at 4°C with capture anti-mouse IFN-γ monoclonal antibody. Plates were washed with PBS and a total of 10 µg/ml of purified Hcp1 in CTL-Medium was added to each well. Plates were allowed to equilibrate for 15 min in the incubator, purified splenocytes were suspended in CTL-Medium with L-glutamine and added to the wells. Plates were incubated for 24 h at 37°C. The plates were washed twice each with PBS and 0.05% Tween-PBS to remove the splenocytes. Next, biotinylated anti-mouse IFN-γ-monoclonal antibody was added, and after 2 h of incubation at room temperature, plates were washed three times with 0.05% Tween-PBS. Streptavidin-alkaline phosphatase conjugate antibody solution was added, and the plates were incubated for 30 min. Following this, plates were washed two times each with 0.05% Tween-PBS and distilled water and developer solution was added according to manufacturer’s recommendations. The reaction was stopped after 15 min by washing the plates three times with distilled water and air-drying overnight. Spots were scanned with an automated ELISpot reader (CTL-Immunospot S6 Analyzer, CTL, Germany) using the ImmunoSpot^®^ software. T-cell responses were quantitated as spot-forming cells (SFC), adjusted to 10^6^ cells per well.

### 2.9 Cytokine/chemokine analysis

Cellular immune responses were determined pre-challenge (27–28 days after the last vaccination) using purified, restimulated splenocytes, or three days after challenge with *B. pseudomallei* K96243 using organ homogenates. The splenocytes were stimulated with 5 µg/ml of Hcp1 for 48 h; organ homogenates obtained post challenge were not stimulated before analysis. Samples were evaluated in Luminex assays using the MAGPIX 36-plex mouse panel (Life Technologies, Grand Island, NY) as per manufacturer directions.

### 2.10 Statistical analyses

All analyses were implemented in SAS version 9.4 (Cary, NC), except as indicated. Survival curves of the vaccinated and control mice were estimated with the Kaplan-Meier method and were compared statistically using the log-rank test with GraphPad Prism 9.0 (San Diego, CA) and SAS. Significant differences in times to death or euthanasia (TTD) at days 7, 21, and 60 after challenge were determined using the Fisher Exact test. The CFU results were summarized as GM (GSD) values, using the LOD/SQRT(2) to replace the values with no recoverable CFU. Likelihood ratio (LR) statistics for type 3 analysis based on negative binomial regression were used for pairwise comparisons between vaccine group CFU. The ELISA data were summarized as the median and interquartile range (Q1, Q3) and the GM (GSE), and pairwise treatment groups were compared by negative binomial generalized linear mixed model; the multiplicity was adjusted by Tukey’s method. ELISpot and Luminex data were log_10_ transformed, and pairwise treatment groups were compared by a linear mixed effects model adjusted by Tukey’s method.

## 3 Results

### 3.1 The protective efficacy of live attenuated and subunit vaccines alone or administered concomitantly against aerosolized *B. pseudomallei*: a preliminary evaluation

**Figure 1:**
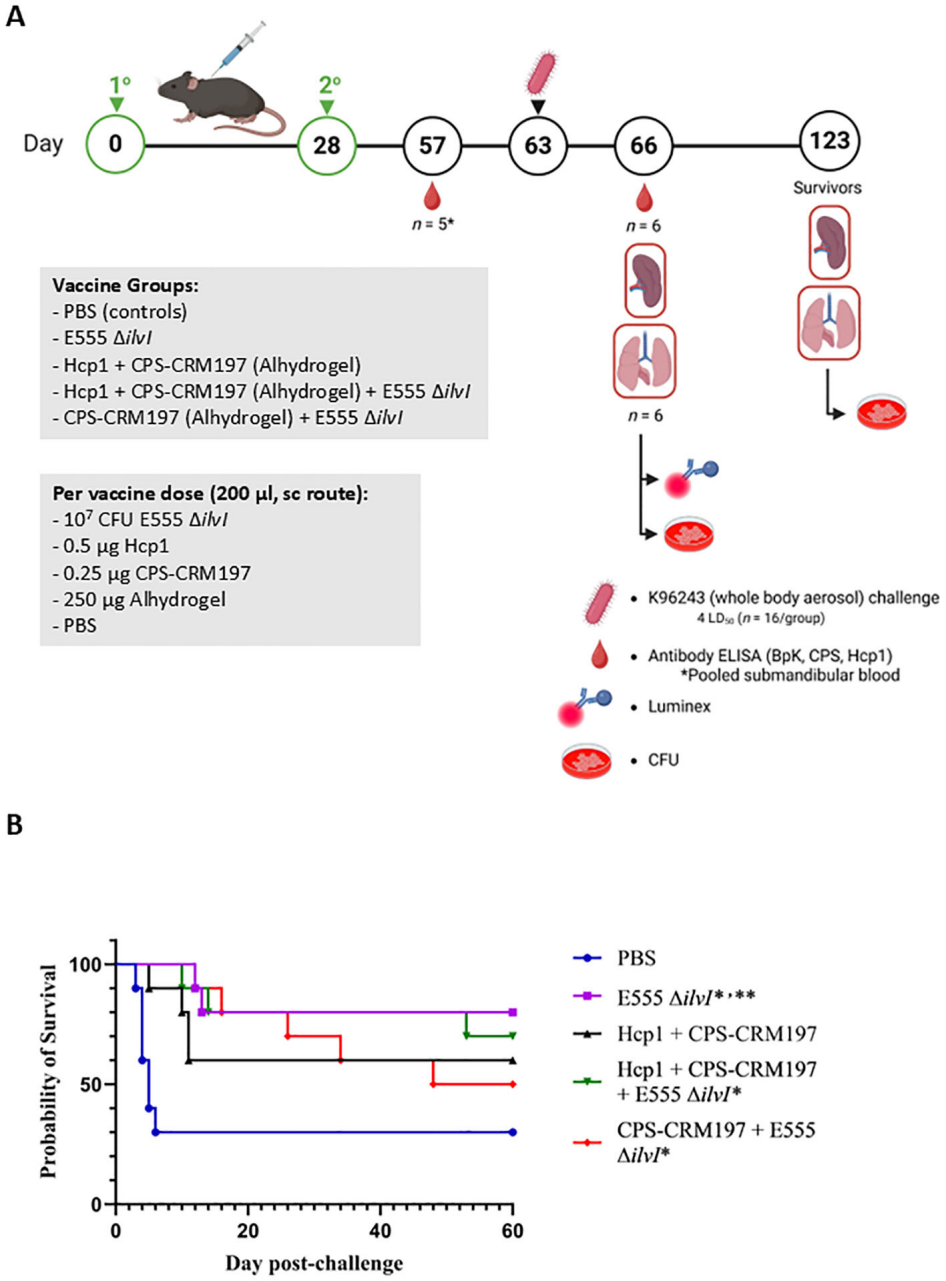
**(A)** Overview of the immunization and challenge strategy for the assessment of live attenuated, subunit and homologous vaccines. Some vaccinated animals were given the live attenuated and subunit vaccines at the same time but at distinct sites (not in the same syringe and the Δ*ilvI* live attenuated strain was delivered in a distinct site than the protein subunit vaccine). The subunit vaccines were delivered with the adjuvant Alhydrogel. The quantities of vaccine components are indicated in the box, and the vaccines were administered twice by the subcutaneous (sc) route, on days 0 and 28. The mice were exposed to *B. pseudomallei* K96243 (4 LD_50_) by the aerosol route day 63. Created in BioRender. Biryukov, S. (2025) https://BioRender.com/otr69lv. **(B)** Survival of homologously vaccinated C57BL/6 mice challenged with whole body aerosol challenges of *B. pseudomallei* K96243 (4 LD_50_, ~1,630 CFU). The mice (*n* = 10/group) were vaccinated with E555 Δ*ilvI* (purple), Hcp1 + CRM197-CPS + Alhydrogel (black), Hcp1 + CRM197-CPS + Alhydrogel + E555 Δ*ilvI* (green) or CRM197-CPS + Alhydrogel + E555 Δ*ilvI* (red), as indicated in **Figure 1A**. Control animals received two doses of PBS (blue). The mice were monitored for 60 days and those that succumbed, or were euthanized, were recorded daily. ^*^The day 21 survival rates of three vaccine groups were greater than that of the PBS controls (*p* = 0.039). ^**^The day 60 survival rate of E555 Δ*ilvI* -vaccinated mice was greater than that of the PBS control group (*p* = 0.035).

A LAV and a defined subunit vaccine were previously shown to provide similar levels of protection in a C57BL/6 mouse model of melioidosis but generated distinct immunological profiles (31). In the current investigation, three different vaccine candidates were evaluated including 1) *B. thailandensis* E555 Δ*ilvI*, 2) CPS-CRM197 adjuvanted with Alhydrogel, and 3) CPS-CRM197 plus Hcp1 adjuvanted with Alhydrogel. The novel LAV strain E555 Δ*ilvI* is a derivative of the CPS-producing strain E555 that harbors a deletion mutation in *ilvI* making it auxotrophic for isoleucine, leucine, and valine, and thereby increases its safety (23, 30, 37). The subunit vaccines contained a CPS-CRM197 glycoconjugate alone or in combination with Hcp1 (33), a T6SS-1 protein that is essential for virulence and is associated with immune responses in survivors of melioidosis (45–48). This study was designed to determine if different combinations of these vaccines would increase protection in mice. **Figure 1A** shows the vaccination and challenge strategies using a homologous vaccination approach with both the prime and boost vaccine formulation being the same.

#### 3.1.1 Humoral immune responses to vaccination

Total IgG responses to the vaccines in mice were assessed using sera collected four weeks after the second dose and about a week before challenge. ELISAs were conducted using the following antigens: irradiated whole cell *B. pseudomallei* K96243 (BpK), CPS, and Hcp1 (**Table 1**; **Supplementary Table 1**). E555 Δ*ilvI* generated a robust IgG response against the BpK, but little to no IgG response against purified CPS or Hcp1 (**Table 1**). The two-component subunit vaccine, Hcp1 + CPS-CRM197, generated a similar IgG response to BpK as E555 Δ*ilvI*, but a much stronger response against CPS and Hcp1. The mean anti-CPS titer of the Hcp1 + CPS-CRM197 group was greatly elevated compared to those of the two subunit vaccine groups which included E555 Δ*ilvI* (*p* ≤ 0.0001 for both). The two combination vaccination approaches, 1) Hcp1 + CPS-CRM197 + E555 Δ*ilvI* and 2) CPS-CRM197 + E555 Δ*ilvI*, generated stronger IgG responses to BpK relative to the homologous subunit vaccine (*p* = 0.005 and *p* = 0.0002, respectively). The mean anti-BpK titer of the combined vaccine group CPS-CRM197 + E555 Δ*ilvI* was also greater than that of the mice vaccinated with E555 Δ*ilvI* alone (*p* = 0.0045). Both subunit vaccine formulations containing Hcp1 generated a strong IgG response to this antigen, *p* < 0.0001 (**Table 1**).

**Table 1:** IgG antibody responses of mice vaccinated with live and subunit vaccines against *B. pseudomallei*.

Vaccine Group^c^	IgG Titer^a,b^					
	BpK		CPS		Hcp1	
	GM	GSE	GM	GSE	GM	GSE
PBS	50	1.00	50	1.00	50	1.00
E555 Δ*ilvI*	38,741	2.21	332	3.87	50	1.00
Hcp1 + CPS-CRM197	27,726	2.65	844,485	1.32	145,876	1.19
Hcp1 + CPS-CRM197 + E555 Δ*ilvI*	116,148	2.53	63,597	2.10	160,000	1.66
CPS-CRM197 + E555 Δ*ilvI*	278,576	1.50	48,503	2.00	50	1.00

a The values represent the GM antibody titer with GSE directed against irradiated whole cell K96243 (BpK), CPS, and Hcp1.

b Blood was collected from 15 mice from each vaccine group one week before challenge. The collections were in pools of three mice each for a total of 5 samples.

c The same vaccine was given for prime and boost. 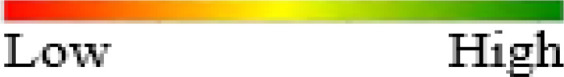

#### 3.1.2 Protection of mice from inhalational melioidosis after vaccination

Five groups of vaccinated C57BL/6 mice were challenged with an approximate dose of 4 LD_50_s of *B. pseudomallei* K96243 by a whole-body aerosol route at 35 days post-boost and survival results are shown in **Figure 1B**. The mice were observed for 60 days post-challenge, and unexpectedly, 30% of the PBS control mice survived challenge (**Figure 1B**). The day 60 results suggest that the most efficacious vaccines were E555 Δ*ilvI* (80% protection) and Hcp1 + CPS-CRM197 + E555 Δ*ilvI* (70% protection); the survival rate of the mice receiving E555 Δ*ilvI* was significantly greater than that of the PBS controls (*p* = 0.035). This preliminary study demonstrated efficacy of the novel *B. thailandensis* E555 Δ*ilvI* vaccine candidate. However, the day 60 mortality rates did not allow us to determine whether combining the LAV and subunit vaccines could increase the protective efficacy against *B. pseudomallei* since there was incomplete mortality in the PBS control group at the completion of the study. Notably, CpG was not included with the subunit vaccines as it had been previously (31) which may have impacted their protective capacity compared to previous studies. Nevertheless, statistical analyses of the survival curves (which reflect differences in times to death or euthanasia [TTD]) by day 21 and day 60 post-challenge, and of survival rates by day 21, further differentiated the groups. By day 21, both the mortality rates and survival curves of all vaccine groups except those receiving Hcp1 + CPS-CRM197 alone were greater than that of the PBS controls (*p* = 0.035 and *p* = 0.010, respectively). By day 60, the survival curves of two vaccine groups were still significantly different from the control curve (*p* = 0.010 for the E555 Δ*ilvI* group and *p* = 0.024 for the Hcp1 + CPS-CRM197 + E555 Δ*ilvI*-vaccinated mice); the survival curve for the Hcp1 + CPS-CRM197 group approached significance with *p* = 0.059. The comparison of proportion succumbing to infection by day 60 was only significant when comparing the E555 Δ*ilvI* group to the PBS control group (*p* = 0.035) (**Figure 1B**; **Supplementary Tables 2A, B**).

#### 3.1.3 Bacteriology

**Figure 2:**
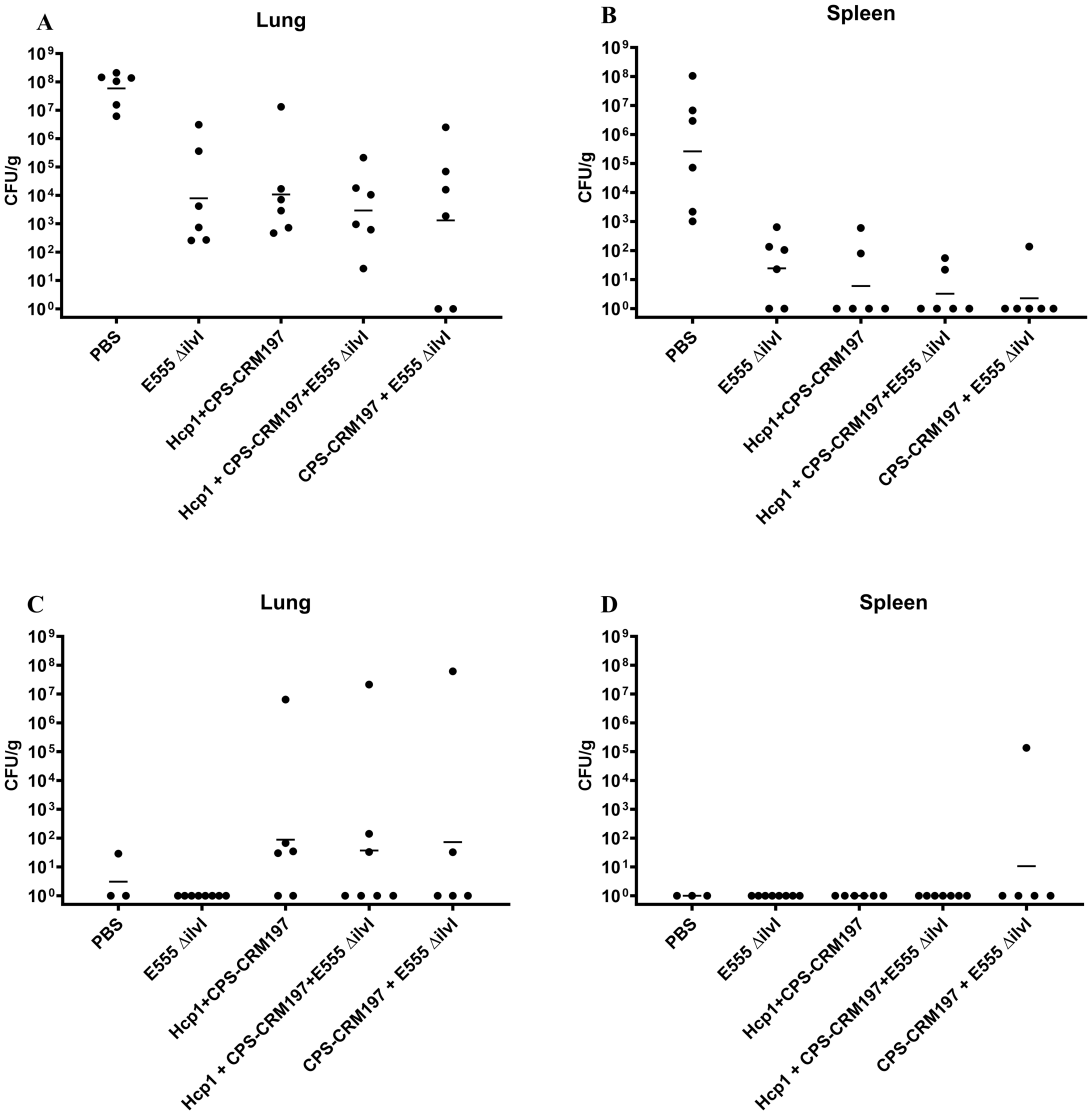
The quantitation of bacterial CFU from tissues of C57BL/6 mice infected with *B. pseudomallei* K96243. The numbers of CFU/g organ were assessed following tissue homogenization, serial dilution and spreading aliquots onto agar medium three days after challenge for lungs **(A)** and spleens **(B)**. The data points represent colony counts for each of six animals per group. Bacterial burdens in the lungs **(C)** and spleen **(D)** of vaccinated mice that survived to the end of study are shown. The horizontal lines are the geometric means.

The numbers of viable bacteria present in the blood, lungs, and spleens were determined on day 3 post-challenge for six animals in each group. Except for one animal in the Hcp1 + CPS-CRM197 + E555 Δ*ilvI* immunized group (1/6 mice were bacteremic), the only mice with detectable bacteria in their blood were four of the six PBS control animals (4/6 mice were bacteremic). As shown in **Figure 2A**; **Supplementary Table 3A**, bacteria were readily detected in the lungs in all groups; however, the four vaccinated groups had GM CFU values which were reduced by approximately 3.5- to 5-log (1.3 to 10.7 x 10^3^ CFU/g) than that of the PBS group (5.89 x 10^7^ CFU/g), with significances ranging from *p* = 0.005 to *p* < 0.0001 compared to the PBS mice. In contrast to the lung samples, spleens (**Figure 2B**) from mice in the three-subunit vaccine-immunized groups exhibited no detectable CFU in four to five of the six samples (*p* < 0.0001 compared to PBS). Most of the samples from the E555 Δ*ilvI*-immunized groups had bacteria in the spleens, albeit low levels (GM of 24.5 CFU/g). The controls had a four log higher GM, 2.63 x 10^5^ CFU/g (*p* < 0.0001 compared to the E555 Δ*ilvI* group). The results suggest that by three days post-challenge the vaccinated animals could clear or reduce dissemination of *B. pseudomallei* more effectively than the unvaccinated control animals.

The number of viable bacteria present in the lungs and spleens of all surviving mice were also determined on day 60 post-challenge. The lungs of all eight surviving E555 Δ*ilvI*-immunized mice had no detectable CFU, whereas some mice in the other four groups had low levels of bacteria (3.1 CFU/g in one of the three surviving PBS mice, and GM values of 37 to 87 CFU/g in the other three vaccine groups, *p* = 0.0001 to *p* = 0.0012 compared to the PBS group (**Figure 2C**; **Supplementary Table 3B**). Except for one mouse, none of the survivors in the five groups had detectable CFU in the spleen; one of five survivors in the CPS-CRM197 + E555 Δ*ilvI* group had 1.36 x 10^5^ CFU/g (the group GM was 11 CFU/g) (**Figure 2D**). Thus, although the vaccines significantly reduced the levels of the challenge strain in the organs at both the early and end of study timepoints compared to unvaccinated controls, except for the E555 Δ*ilvI* group, sterile immunity was not achieved.

#### 3.1.4 Cell-mediated immune responses.

Cellular immune responses were evaluated three days after challenge with *B. pseudomallei* K96243 in the lung homogenates from control and vaccinated mice (**Table 2**; **Supplementary Table 4**). Relative to the PBS controls, the mice immunized with E555 Δ*ilvI* alone displayed two-fold greater levels of IFN-γ in the lung homogenates at 3 days post-challenge, while the IFN-γ level of the Hcp1 + CPS-CRM197 group was lower than the controls (*p* = 0.001) and the other vaccine groups (*p* < 0.0001). IFN-γ is vital for host defense against *B. pseudomallei* infections; the basis for the reduced levels of IFN-γ in the group vaccinated with the Hcp1 + CPS-CRM197 conjugate alone is not clear but might be related to the absence of the CpG immunostimulant in this experiment (31). In contrast to the others, this group also exhibited depressed levels of IL-18 and IL-22 (*p* < 0.0001 and *p* = 0.0003, respectively, when compared to the PBS controls), which are involved in co-stimulation of IFN-γ and reduction of lung inflammation, respectively (49–51). This may also be partially attributed to the greater suppression of IL-17A in this vaccine group relative to others. As shown in **Table 2**, animals that received any of the four vaccines displayed reduced levels of many pro-inflammatory cytokines, such as IL-1β, MIP-2α, IL-6, MIP-1α, MIP-1β, GRO-α, MCP-1, G-CSF, LIF, IL-1α, GM-CSF, TNF-α, IL-17A, and MCP-3 (*p* = 0.0421 to *p* < 0.0001). Spleen homogenates were also evaluated, and the relative responses of the vaccine groups compared to the controls were similar to those of the lung (**Supplementary Table 5**). Depressed responses were observed for many cytokines in all four vaccine groups, such as G-CSF, IL-6, GRO-α, MCP-1, IL-1β, IL-13, MCP-3, LIF, or IL-1α (*p* = 0.0042 to *p* < 0.0001, **Supplementary Tables 4**, **5**). However, the IFN-γ levels were either not statistically different (the E555 Δ*ilvI* group) or significantly reduced (the remaining vaccine groups, *p* = 0.0003 to *p* < 0.0001) relative to the PBS controls; and IL-17A levels in these four groups were also not significantly different compared to controls. These differences in cytokine results in the spleen compared to the lung might be attributed to the early stage after aerosol challenge with limited systemic bacterial dissemination.

**Table 2:** The cytokine responses in lung homogenates obtained three days after challenge with aerosolized *B. pseudomallei* K96243.

Cytokine^a^	E555 Δ*ilvI*	Hcp1 + CPS-CRM197	CPS-CRM197 + E555 Δ*ilvI*	Hcp1 + CPS-CRM197 + E555 Δ*ilvI*
IFNγ	*2.04*	**0.27**	*1.47*	*1.47*
IL-4	*1.05*	**0.58**	*1.21*	*1.19*
IL-22	*1.04*	**0.35**	*0.97*	*0.99*
IL-2	*1.03*	*0.82*	*0.99*	*1.03*
RANTES (CCL5)	*1.02*	*0.94*	*1.03*	*1.02*
IL-28	*0.97*	*0.73*	*0.98*	*1.02*
IL-12p70	*0.96*	**0.53**	*0.93*	*0.87*
IL-5	*0.94*	**0.58**	*1.26*	*1.12*
IL-23	*0.87*	*0.65*	*0.95*	*1.05*
IL-9	*0.86*	*0.79*	*0.99*	*1.01*
IL-15	*0.85*	*0.82*	*0.79*	*0.78*
IL-13	*0.84*	*0.53*	*1.23*	*1.03*
IL-18	*0.79*	**0.30**	*0.76*	*0.71*
IL-27	*0.71*	**0.54**	**0.47**	**0.55**
IP-10 (CXCL10)	*0.67*	**0.37**	*0.63*	*0.59*
IL-3	*0.53*	**0.43**	*0.87*	*0.70*
Eotaxin	**0.52**	**0.38**	**0.39**	**0.40**
IL-31	*0.51*	**0.47**	*0.66*	*0.55*
MCP-3 (CCL7)	**0.36**	**0.15**	**0.24**	**0.22**
M-CSF	**0.32**	*0.39*	**0.31**	**0.28**
IL-17A (CTLA-8)	**0.31**	**0.16**	**0.41**	**0.33**
TNF-α	**0.19**	**0.10**	**0.18**	**0.14**
GM-CSF	**0.19**	**0.12**	**0.20**	**0.18**
IL-1α	**0.19**	**0.15**	**0.18**	**0.15**
LIF	**0.14**	**0.08**	**0.12**	**0.10**
G-CSF	**0.13**	**0.08**	**0.10**	**0.06**
MCP-1 (CCL2)	**0.12**	**0.05**	**0.07**	**0.07**
GRO-α (CXCL1)	**0.11**	**0.04**	**0.07**	**0.03**
MIP-1β (CCL4)	**0.09**	**0.07**	**0.09**	**0.05**
MIP-1α (CCL3)	**0.06**	**0.05**	**0.06**	**0.04**
IL-6	**0.04**	**0.02**	**0.04**	**0.03**
MIP-2α (CXCL2)	**0.03**	**0.02**	**0.03**	**0.01**
IL-1β	**0.02**	**0.01**	**0.02**	**0.01**

**^a^** The cytokine results are shown as the ratio to PBS, and are based on the Geometric Mean (pg/ml). Italicized and not bolded (Not Significant), Bolded (*p* ≤ 0.05). 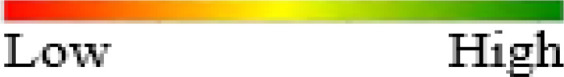

### 3.2 The protective efficacy of vaccine candidates delivered by homologous and heterologous vaccination strategies against aerosol exposure to *B. pseudomallei*

**Figure 3:**
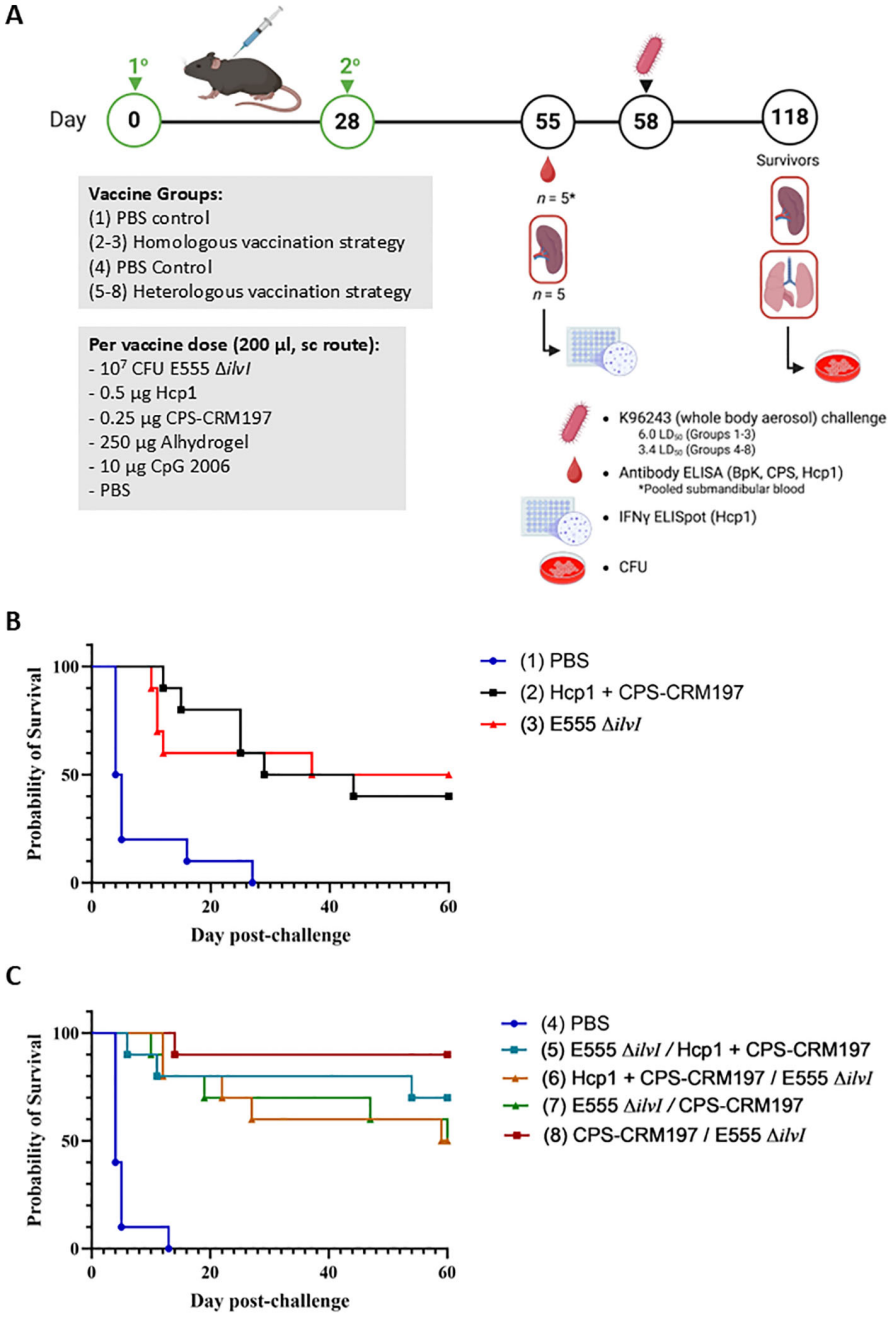
**(A)** Overview of the immunization and challenge strategy for the assessment of *Burkholderia* vaccine candidates delivered via homologous and heterologous vaccination strategies. The vaccines were administered twice by sc route, on days 0 and 28, and the mice were exposed to *B. pseudomallei* K96243 (6.0 LD_50_ to groups 1–3 and 3.4 LD_50_ to groups 4 - 8) by the aerosol route four weeks later; *n* = 15 mice/group. As detailed in **Table 3**, groups 2 and 3 received homologous vaccines for the prime and boost doses; groups 5–8 received the heterologous vaccines; and groups 1 and 4 included the PBS controls. The subunit vaccines were delivered with the adjuvant Alhydrogel and immunostimulant CpG 2006 (10 µg). Created in BioRender. Biryukov, S. (2025) https://BioRender.com/oji8j26. **(B)** Survival curves of vaccinated C57BL/6 mice challenged with whole body aerosol challenges of *B. pseudomallei* K96243 (6.0 LD_50_, ~2,400 CFU). The mice (*n* = 10/group) received a prime and boost with the same vaccine (homologous), as shown in **Table 3**. The subunit vaccines included Alhydrogel and the CpG immunostimulant. Control animals received two doses of PBS. The mice were monitored for 60 days and those that succumbed to infection, or were euthanized, were recorded daily. **(C)** Survival curves of vaccinated C57BL/6 mice challenged with whole body aerosol challenges of *B. pseudomallei* K96243 (3.4 LD_50_, ~1,360 CFU). The mice (*n* = 10/group) received heterologous vaccinations in which the prime and boost were different vaccines (separated by “/” in the legend), as shown in **Table 3**. The subunit vaccines included Alhydrogel and CpG. Control animals received two doses of PBS. The mice were monitored for 60 days and those that succumbed to infection, or were euthanized, were recorded daily.

An expanded evaluation of various vaccines was conducted to assess the efficacy of homologous and heterologous vaccination strategies with LAV E555 Δ*ilvI* and the subunit vaccine Hcp1 + CPS-CRM197 (31, 33). All subunit vaccines described were delivered with Alhydrogel and CpG, except where indicated (**Table 3**). The homologous vaccine strategy used the same vaccine candidate for the prime and boost (groups 2 and 3, and group 1 controls), while the heterologous vaccine strategy utilized different vaccine candidates for the prime and boost (groups 5 – 8, and group 4 controls). The strategies for immunization and challenge were similar to that illustrated in **Figure 1A** and are shown in **Figure 3A**.

**Table 3:** The *B. pseudomallei* vaccine candidates evaluated in homologous and heterologous vaccine strategies.

Vaccination strategy^a,b^	Vaccine group #	Prime	Boost
Homologous	1	PBS	PBS
	2	Hcp1 + CPS-CRM197	Hcp1 + CPS-CRM197
	3	E555 Δ*ilvI*	E555 Δ*ilvI*
Heterologous	4	PBS	PBS
	5	E555 Δ*ilvI*	Hcp1 + CPS-CRM197
	6	Hcp1 + CPS-CRM197	E555 Δ*ilvI*
	7	E555 Δ*ilvI*	CPS-CRM197
	8	CPS-CRM197	E555 Δ*ilvI*
	9	CPS-CRM197, **no** CpG	E555 Δ*ilvI*

a All subunit vaccines contained Alhydrogel and CpG, except where indicated.

b Number of mice: *n* = 15, 5 for immunology and 10 for challenge, with the exception of group 9 which only had 10 mice for challenge.

#### 3.2.1 Humoral immune responses to vaccination

Total IgG responses to the vaccines were assessed using blood collected 27 days after the boost dose, prior to challenge in vaccinated mice. ELISAs were conducted using the BpK, CPS, and Hcp1 antigens. The total serum IgG titers following homologous vaccination of mice were determined, as shown in **Table 4A**. The GM titer to the BpK antigen produced by group 2 (Hcp1 + CPS-CRM197) was nearly twice that of the anti-BpK titer elicited by the E555 Δ*ilvI* vaccine. Nevertheless, both vaccine groups produced anti-BpK titers that were significantly greater than that of the PBS controls (*p* = 0.0006 and *p* = 0.0015, for groups 2 and 3, respectively). Antibody titers to CPS were high in group 2 and were negligible in the other two groups, as expected (*p* < 0.0001 comparing group 2 results to those of the other two groups). Only the vaccine containing Hcp1 produced significant responses to this antigen (*p* = 0.0012 compared to groups 1 and 3). Thus, overall, the E555 Δ*ilvI* vaccine stimulated the lowest antibody levels with very low titers to BpK and no detectable antibodies to purified CPS and Hcp1; these results were similar to those described above in **Table 1**. The findings suggest that there may be substantial surface antigen differences between *B. pseudomallei* and *B. thailandensis*, even though E555 Δ*ilvI* produces a *B. pseudomallei*-like CPS.

**Table 4:** Total IgG antibody responses of mice vaccinated with live and subunit vaccines against *B. pseudomallei*: Strategy Comparisons.

A - Homologous vaccinations^a^								
Vaccine group #	Prime	Boost	IgG Titer^b^ ^,^ ^c^					
			Bpk		CPS		Hcp1	
			GM	GSE	GM	GSE	GM	GSE
1	PBS	PBS	50	1.00	50	1.00	50	1.00
2	Hcp1 + CPS-CRM197	Hcp1 + CPS-CRM197	12,126	2.38	147,033	2.17	40,445	5.60
3	E555 Δ*ilvI*	E555 Δ*ilvI*	6,390	2.68	50	1.00	50	1.00

B - Heterologous vaccinations^a^								
Vaccine group #	Prime	Boost	IgG Titer^b^ ^,^ ^c^					
			Bpk		CPS		Hcp1	
			GM	GSE	GM	GSE	GM	GSE
4	PBS	PBS	50	1.00	50	1.00	50	1.00
5	E555 Δ*ilvI*	Hcp1 + CPS-CRM197	1,668	2.06	22,111	1.90	27,902	2.85
6	Hcp1 + CPS-CRM197	E555 Δ*ilvI*	1,388	3.62	12,699	1.83	140,394	1.17
7	E555 Δ*ilvI*	CPS-CRM197	797	1.70	11,596	2.37	50	1.00
8	CPS-CRM197	E555 Δ*ilvI*	958	2.32	8,392	4.09	50	1.00

a All vaccines containing subunit antigens included Alhydrogel and CpG.

b Blood was collected from 15 mice from each group before challenge. The collections were in pools of three mice each for a total of 5 samples.

c The values represent the GM antibody titer with GSE directed against irradiated whole cell K96243 (BpK), CPS, and Hcp1. 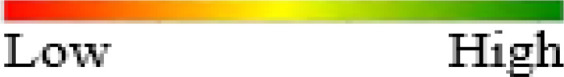

In addition to total IgG titers, IgG1 and IgG2c responses to the irradiated whole-cell BpK antigen were assessed for the homologous vaccination groups (**Supplementary Table 6A**). The group 2 subunit vaccine induced IgG1 titers to BpK antigen that were much higher than the corresponding IgG2c levels (IgG2c/IgG1 GM ratio of 0.08). In contrast, E555 Δ*ilvI* skewed titers toward IgG2c rather than IgG1 (IgG2c/IgG1 GM ratio of 6.99).

The IgG levels following heterologous vaccination of mice are shown in **Table 4B**. Groups 5 and 6, in which the LAV and Hcp1 + CPS-CRM197 were given as the prime and boost, or in the reverse order, exhibited titers to the BpK antigen that were the highest, *p* = 0.0371 and *p* = 0.0506 versus the controls, respectively. Elevated titers to CPS were elicited by all four vaccine groups (*p* = 0.0004 to *p* = 0.0027 compared to group 4), each of which included the CPS-CRM197 subunit. The anti-CPS titers were not significantly associated with the order of antigen delivery, i.e., prime or boost (**Table 4B**); however, boosting with CPS-CRM197 appeared to favor stimulation of greater anti-CPS titers compared to priming with the subunit formulation. High GM levels of anti-Hcp1 antibodies were observed when the two vaccines containing this antigen were used in groups 5 and 6 (*p* < 0.0001 compared to groups 4, 7, and 8). The higher level was elicited by group 6, which were immunized with the vaccine containing Hcp1 in the prime dose. It appears that the order of delivery of the vaccines potentially impacted the antibody responses to CPS and Hcp1, while no discernable differences were observed against BpK.

All four heterologous vaccine strategies stimulated IgG1 and IgG2c antibodies to the BpK antigen (**Supplementary Table 6B**). Groups 5–7 exhibited IgG2c/IgG1 ratios that were closely balanced, i.e., not Th1- or Th2-skewed (ratios of 0.69, 0.63, and 0.87, respectively). In contrast, the group 8 elicited a negligible level of IgG2c antibodies and its IgG2c/IgG1 ratio was 0.12. Thus, the subclass IgG responses of these mice, which were vaccinated with a prime dose of CPS-CRM197 in the absence of Hcp1, were Th2-skewed. All the heterologous vaccine combinations, regardless of the order of delivery (whether CPS-CRM197 conjugate was in the prime or the boost dose [groups 5 – 8]) elicited low anti-BpK titers, yet they were associated with survival rates of 50 – 90% (**Table 4B**, **Figure 3C**). This included one of the most protective vaccine formulations and regimens (CPS-CRM197 prime followed by a E555 Δ*ilvI* boost) which induced a very low antibody titer to whole cell BpK antigen (**Table 4B**). There was no apparent association between pre-challenge anti-BpK titers elicited by the homologous vaccines and the extent of protection.

#### 3.2.2 Protection of mice from inhalational melioidosis after vaccination

The various groups of vaccinated and control mice (*n* = 10/group) described in **Table 3** were challenged by the aerosol route with *B. pseudomallei* K96243 on day 58 (**Figure 3A**). Homologously vaccinated mice received 2,400 CFU (approximately 6.0 LD_50_s) (**Figure 3B**) and heterologously vaccinated mice received 1,360 CFU (approximately 3.4 LD_50_s) (**Figure 3C**). The highest level of protection afforded by a homologous vaccine at day 60 post-challenge was conferred by a prime and boost of the LAV E555 Δ*ilvI* (group 3, 50%) (**Figure 3B**), while the highest level of protection by a heterologous vaccine was produced by administering a CPS-CRM197 prime followed by a E555 Δ*ilvI* boost (group 8, 90%), as shown in **Figure 3C**. Statistical analyses of the data further clarified these conclusions, as detailed below.

By 21 days post-challenge, the two homologously vaccinated groups exhibited greater survival rates compared to that of the PBS control mice, i.e., *p* = 0.006 for group 2 (Hcp1 + CPS-CRM197) and 0.057 for group 3 (E555 Δ*ilvI*) (**Figure 3B**). Also, the mean TTDs at day 21 of both homologous vaccination groups were significantly greater than that of the PBS group (14.7 and 11.6 days and 6.7 days, respectively, *p* ≤ 0.0032). At the study endpoint (60 days post challenge), only the group 3 LAV-immunized mice exhibited a significantly greater survival rate compared to the PBS group (*p* = 0.033) (**Figure 3B**) while the survival rates of groups 2 and 3 were not statistically different. As observed at day 21, the TTDs at day 60 indicated that both vaccines extended the mean TTD compared to the controls (*p* = 0.0002 and *p* = 0.0006, respectively). The mean TTD of the controls was 7.8 days compared to 32.6 and 26.6 days for the groups 2 and 3, respectively. The results suggested that the LAV and Hcp1 + CPS-CRM197 subunit vaccines were efficacious, albeit neither was completely protective. Also, since differences in survival rates of the two vaccine groups could not be statistically distinguished, these results supported the evaluation of a heterologous vaccine scheme.

Four groups of mice were given heterologous prime and boost doses of vaccine, as shown in **Table 3** and **Figure 3C**. By 21 days post-challenge, the ten control mice had succumbed, and all the vaccine groups exhibited greater survival rates than the controls (*p* = 0.0031 to *p* = 0.0001). Additionally, all four vaccine groups also had significantly extended mean TTDs at day 21 post-challenge compared to the control mice (*p* < 0.0001). Analyses of the survivors at the study endpoint (60 days post-challenge), showed that the four vaccines were more protective compared to the controls, with survival rates ranging from 50% to 90% (**Figure 3B**), with *p* = 0.0325 to *p* = 0.0001. Group 8 (prime CPS-CRM197/boost E555 Δ*ilvI*) had the highest level of survival at day 60 (90%), which was significantly greater than the PBS group 4 (*p* < 0.0001). However, the three vaccines associated with 50% to 70% protection (groups 5, 6, and 7) were not statistically different compared to the mice receiving the prime CPS-CRM197/boost E555 Δ*ilvI* vaccination strategy. The mean TTDs at day 60 again indicated that all four vaccines significantly extended the mean TTD compared to the controls (*p* < 0.0001).

Since the *B. pseudomallei* challenges of the homologous and the heterologous vaccine groups (**Figure 3**) were performed on the same day, we compared the protection afforded across all eight groups. The survival rate of group 8 at 60 days post-challenge (90%) approached significance relative to the survival of homologous vaccine group 2, which induced 40% protection, with *p* = 0.057; but less when compared to the homologous vaccine that protected 50% of group 3 (E555 Δ*ilvI*). Thus, the heterologous vaccine scheme identified a combination which was 90% protective; and all four heterologous vaccines protected ≥ 50% of mice, findings which were comparable to those of the homologous vaccines. Importantly, however, the challenge dose of the heterologously vaccinated mice was roughly half of the homologously vaccinated mice (3.4 versus 6.0 LD_50_).

#### 3.2.3 Cell-mediated immune responses

**Figure 4:**
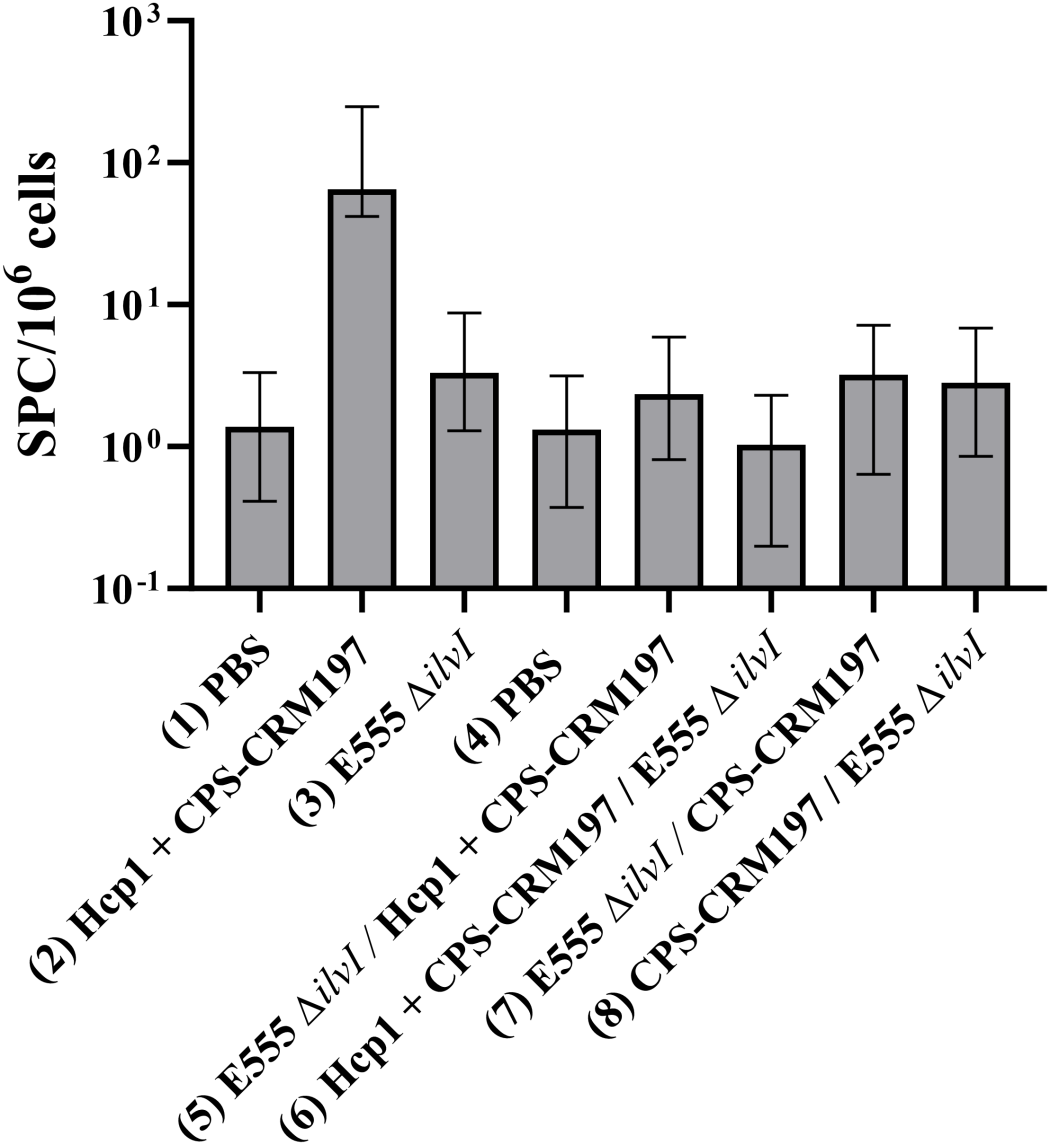
IFNγ-secreting splenocytes obtained from control and vaccinated C57BL/6 mice twenty-seven days post boost following stimulation with 10 µg/ml Hcp1. The splenocyte response of mice (*n* = 5/group) was assessed as spot forming cells (SFC), adjusted to 10^6^ cells per well. Values represent the GM ± the GSE.

The cellular immune responses of vaccinated mice were first evaluated using ELISpot assays to measure IFN-γ secretion by splenocytes restimulated with Hcp1. Controls were incubated with medium alone and resulted in low levels of IFN-γ-secreting cells in all groups (data not shown). As illustrated in **Figure 4**, the highest numbers of IFN-γ-secreting splenocytes after Hcp1 stimulation were produced by the homologous vaccine group 2 (*p* < 0.0001 compared to the PBS control group 1). The responses of homologous group 3 and the four heterologous vaccine groups were not significantly different from the control groups 1 or 4, and were less than those of homologous group 2, *p* < 0.0001 compared to groups 5-8.

### 3.3 The protective efficacy of selected homologous and heterologous vaccination schemes against *B. pseudomallei* challenges

**Figure 5:**
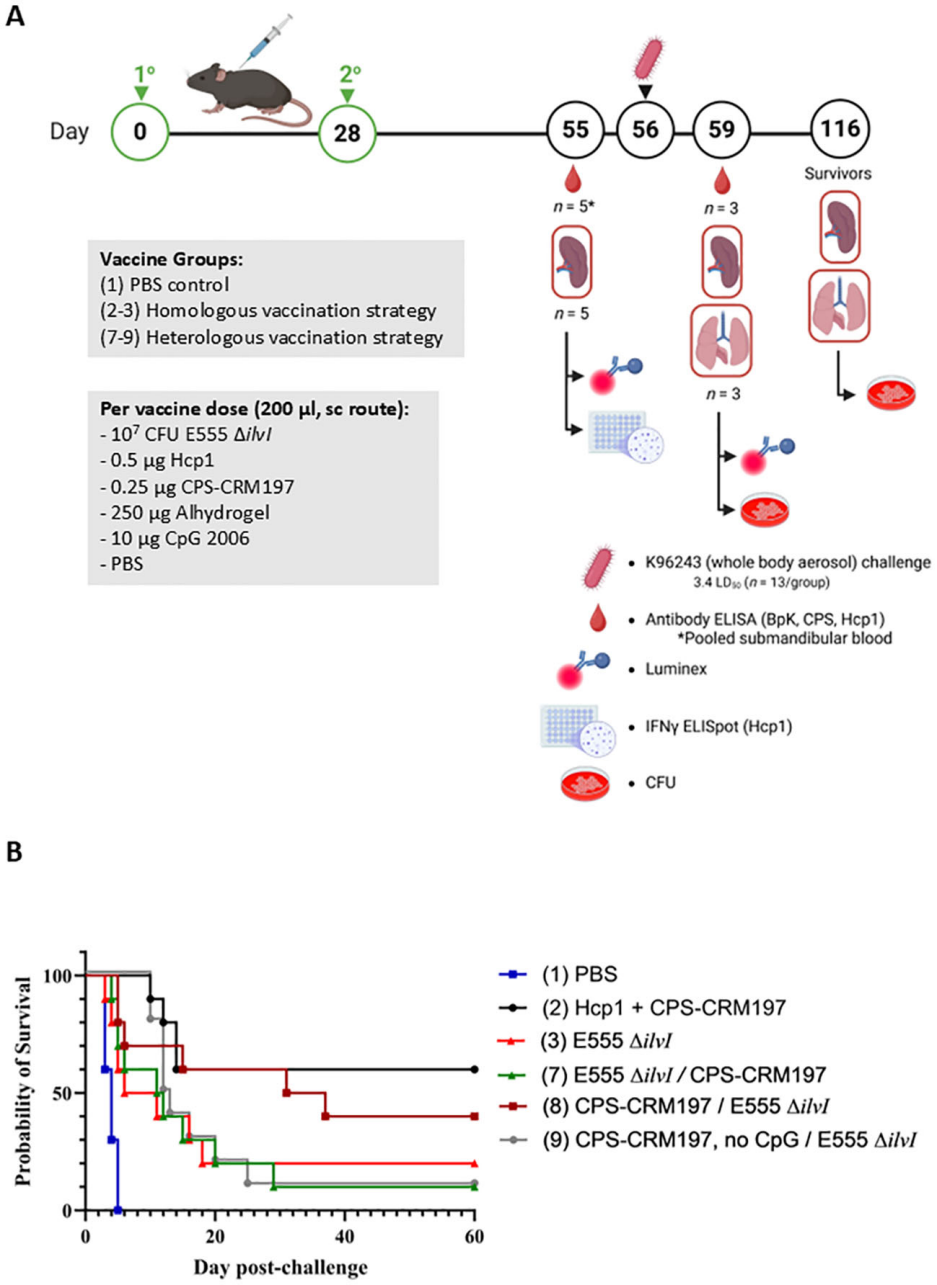
**(A)** Overview of the immunization and challenge strategy for the assessment of *Burkholderia* vaccine candidates delivered via homologous and heterologous vaccination strategies. The vaccines were administered twice by sc route, on days 0 and 28; *n* = 18 mice/group. Mice were exposed to *B. pseudomallei* K96243 (3.4 LD_50_) by the aerosol route four weeks later; *n* = 13 mice/group. As detailed in **Table 3**, groups 2 and 3 received homologous vaccines for the prime and boost doses; groups 7, 8, and 9 received the heterologous vaccines; and group 1 included the PBS controls. The subunit vaccines were delivered with the adjuvant Alhydrogel and, except for group 9, the immunostimulant CpG. Created in BioRender. Biryukov, S. (2025) https://BioRender.com/rfgku3j. **(B)** Survival of vaccinated C57BL/6 mice challenged with whole body aerosol challenges of *B. pseudomallei* K96243 (3.4 LD_50_s, ~1,610 CFU). The heterologous and homologous vaccine groups (*n* = 10 each) are listed in **Table 3**. The subunit vaccines included Alhydrogel and CpG, except group 9 as shown in **Table 3**. The mice were monitored for 60 days and those that succumbed to infection, or were euthanized, were recorded daily. For heterologous vaccine groups 7, 8, and 9 the different prime and boost formulations are separated by “/” in the legend.

To ensure reproducibility, additional evaluation of the LAV and subunit vaccine candidates was performed using selected homologous and heterologous vaccination strategies that demonstrated promising degrees of protection. Two homologous and three heterologous vaccination strategies, corresponding to groups 2 and 3 (homologous strategy) and groups 7 - 9 (heterologous strategy) (**Table 3**) were re-evaluated. Each group received a prime and boost dose of vaccine, or PBS alone (group 1). All except one subunit vaccine included both Alhydrogel and CpG. To evaluate the importance of CpG in the heterologous prime/boost strategy, group 9 which contained Alhydrogel-only was included. The scheme for vaccination, challenge, and collection (**Figure 5A**) was aligned with the previous studies (**Figures 1A**, **3A**).

#### 3.3.1 Humoral immune responses to vaccination

Antibody responses to the vaccine constituents (CPS and Hcp1) were evaluated as potential correlates of protection (**Table 5**). Except for the homologous LAV group, all vaccines stimulated significant titers to the CPS (*p* < 0.0001 when compared to controls) with the highest levels being produced by the group receiving two doses of Hcp1 + CPS-CRM197 (group 2). In agreement with **Table 4**, anti-Hcp1 antibodies were only elicited in the vaccine containing this antigen (group 2).

**Table 5:** Total IgG antibody responses of mice vaccinated with selected vaccine strategies against *B. pseudomallei*.

Vaccine group #	Prime^a^	Boost^a^	IgG Titer^b^ ^,^ ^c^					
			BpK		CPS		Hcp1	
			GM	GSE	GM	GSE	GM	GSE
1	PBS	PBS	50	1.00	50	1.00	50	1.00
2	Hcp1 + CPS-CRM197	Hcp1 + CPS-CRM197	3,330	3.41	168,630	2.02	885,824	1.63
3	E555 Δ*ilvI*	E555 Δ*ilvI*	553	2.07	50	1.00	50	1.00
7	E555 Δ*ilvI*	CPS-CRM197	877	1.72	4,196	1.72	50	1.00
8	CPS-CRM197	E555 Δ*ilvI*	1,213	2.01	23,193	2.02	50	1.00
9	CPS-CRM197, **no** CpG	E555 Δ*ilvI*	1,213	2.24	80,635	1.18	50	1.00

a All vaccines containing subunit antigens included Alhydrogel and CpG, except for group 9 (had Alhydrogel alone).

b Blood was collected from 15 mice/group one week before challenge. The collections were in pools of three mice each for a total of 5 samples.

c The values represent the GM antibody titer with GSE directed against irradiated whole cell K96243 (BpK), CPS, and Hcp1. 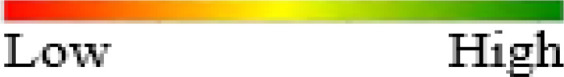

As shown in **Table 5**, four vaccine groups exhibited titers to the BpK antigen that were significantly greater than that of the control group (*p* = 0.0003 to *p* = 0.0295). As seen in the earlier experiments, the LAV homologous vaccine (group 3) elicited the lowest anti-BpK titer (*p* = 0.0588 compared to PBS) as well as low IgG1 and IgG2c titers to BpK (**Table 6**). Except for group 3, all vaccines induced higher levels of IgG1 compared to IgG2c anti-BpK antibodies. While the IgG2c/IgG1 ratio of the LAV group (2.40) was highly Th1 polarized (albeit both titers were low), the ratios of the other groups, especially group 9, were distinctly Th2-skewed (IgG2c/IgG1 ratios of 0.01 to 0.14). These findings were again in agreement with those of the two studies described above (**Supplementary Tables 6A, B**).

**Table 6:** The subclass antibody titers to the BpK antigen of mice vaccinated with selected vaccine strategies.

Vaccine group #	Prime^a^	Boost^a^	IgG1^b^ ^,^ ^c^		IgG2c^b^ ^,^ ^c^		IgG2c / IgG1 ratio
			GM	GSE	GM	GSE	
1	PBS	PBS	50	1.00	50	1.00	1.00
2	Hcp1 + CPS-CRM197	Hcp1 + CPS-CRM197	6,682	3.96	918	3.22	0.14
3	E555 Δ*ilvI*	E555 Δ*ilvI*	50	1.00	120	1.50	2.40
7	E555 Δ*ilvI*	CPS-CRM197	877	2.58	95	1.91	0.11
8	CPS-CRM197	E555 Δ*ilvI*	1,106	3.21	57	1.10	0.05
9	CPS-CRM197, **no** CpG	E555 Δ*ilvI*	6,390	2.21	50	1.00	0.01

a All vaccines containing subunit antigens included Alhydrogel and CpG, except for group 9 (had Alhydrogel alone).

b Blood was collected from 15 mice per group one week before challenge. Collections were in pools of three mice each for a total of 5 samples.

c The values are the GM anti-BpK antibody tites and GSE. 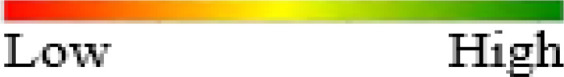

#### 3.3.2 Protection of mice from inhalational melioidosis after vaccination

To assess vaccine efficacy, six groups (groups 1, 2, 3, 7, 8 and 9) of C57BL/6 mice (*n* = 13 mice) were challenged with 3.4 LD_50_s of *B. pseudomallei* K96243 via whole-body aerosol four weeks after the second immunization (day 56). Three mice from each group were euthanized three days post-challenge and tissues were cultured to compare the relative bacterial burdens at this early timepoint. The remaining ten mice were observed for 60 days post-challenge and survival results are shown in **Figure 5B**. All the PBS control mice in group 1 succumbed to disease or were euthanized in accordance with early endpoint euthanasia criteria by day 5 (**Figure 5B**) whereas the five vaccines all provided an extended TTD compared to the PBS control group.

By 21 days post-challenge, two groups (2 and 8) exhibited the highest survival rates (60%), which was significantly higher than that of the PBS controls (*p* = 0.011). In contrast, the survival rates of groups 3, 7, and 9 (20%) were not statistically greater than the controls. The mean TTDs by day 21 of all five vaccine groups were significantly extended compared to the controls (means of 3.9 days for PBS and 10.4 – 14.5 days for the vaccinated mice, *p* = 0.003 to *p* < 0.0001). At the end of the 60-day period, the highest survival rates were observed in homologous vaccination group 2 (60%, *p* = 0.011 *vs* group 1 controls) and group 8 (40%). Of the three heterologous groups, the group 8 vaccine was the most protective, whereas only 10% of the group 7 and 9 mice survived until day 60. None of the survival rates for any of the vaccine groups were statistically different from one another. Nonetheless, the mean TTDs of all five vaccine groups was greater than that of the controls at the study endpoint, (*p* = 0.003 for group 3, *p* = 0.0004 for group 7, and *p* < 0.0001 for the other three groups).

#### 3.3.3 Bacteriology

**Figure 6:**
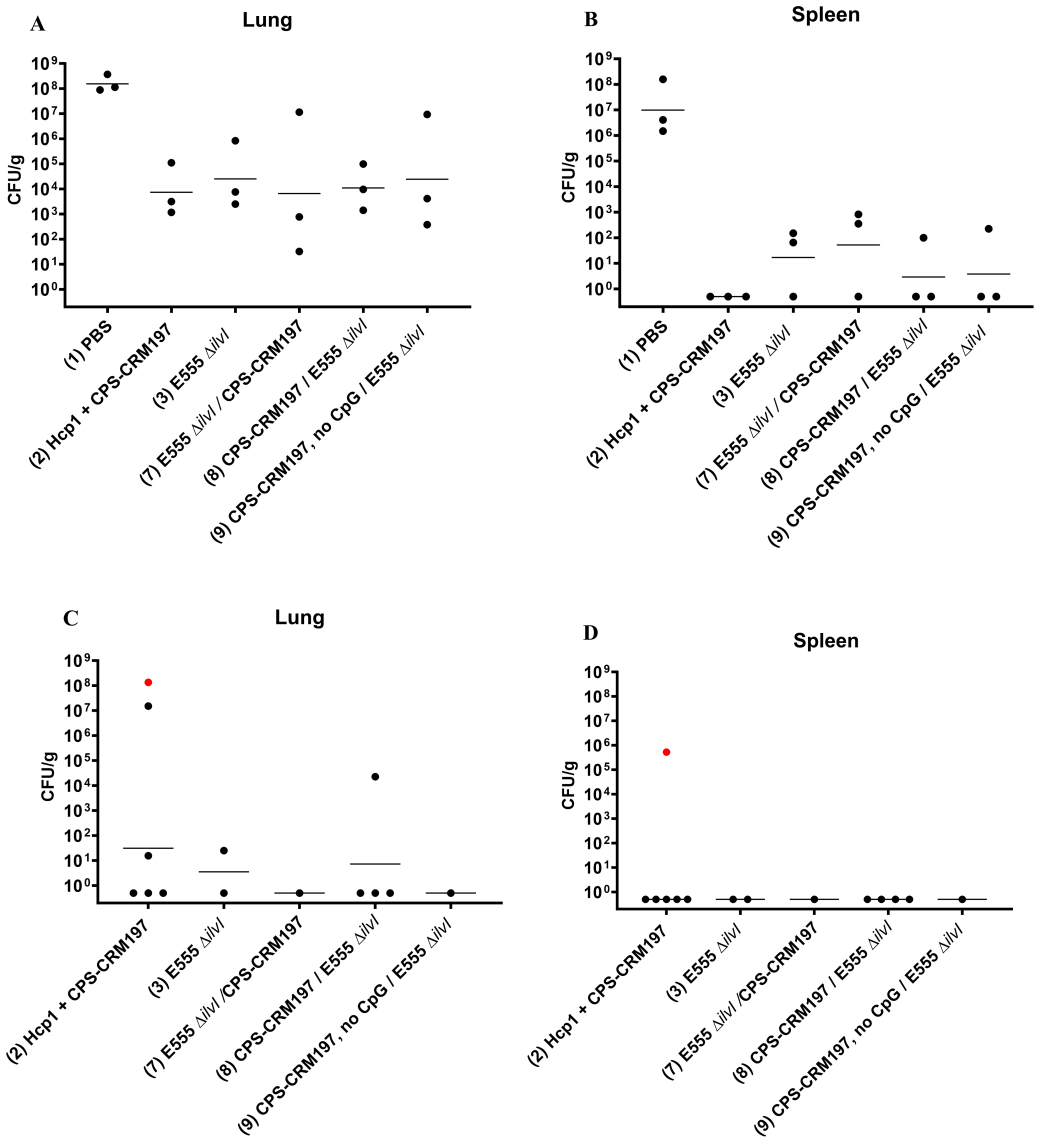
Bacterial burdens in the blood and organs of vaccinated mice infected with *B. pseudomallei* K96243. The numbers of CFU/g organ were assessed following tissue homogenization, serial dilution and spreading aliquots onto agar medium three days after challenge for lungs **(A)** and spleens **(B)**. The data points represent colony counts for each of three animals per group. Bacterial burdens in the lungs **(C)** and spleens **(D)** of vaccinated mice that survived to the end of study are shown, all spleens were negative for *B. pseudomallei.* None of the PBS control mice survived. The horizontal lines are the GM. One mouse was found dead the day of necropsy and was shown to be positive for *B. pseudomallei* in both lungs and spleen [shown as red data points in panels (**C, D**)] but was omitted from the GM calculation because of the time between death and sample collection was undetermined.

To determine bacterial loads in tissues, three mice from each group were euthanized three days after challenge, and lungs, spleens, and blood were cultured. All the vaccines assessed prevented bacteremia (*p* = 0.001) versus PBS controls, which were bacteremic. Vaccination was associated with a nearly 4-log GM CFU reduction in bacteria in the lungs (**Figure 6A**) and resulted in a ≥ 4-log reduction in bacterial dissemination to the spleens (*p* = 0.0044 to *p* = 0.0005) compared to controls (**Figure 6B**). No bacteria were recovered from any of the spleens obtained from group 2. The GM CFU in the lungs were significantly less than that of the controls for groups 2, 3, and 8 (*p* = 0.0001 to *p* = 0.003), almost significantly less for group 9 (*p* = 0.052), but not for group 7 (*p* = 0.088 compared to the PBS controls).

The number of viable bacteria present in the spleens and lungs from all surviving mice were also determined at end of study post challenge. A mouse succumbed to *B. pseudomallei* infection on the day of necropsy and was cultured (depicted as red data points), however these data were omitted from GM calculations due to the period between death and sample collection. All but one spleen from surviving vaccinated mice were sterile (limit of detection 5 CFU/spleen) and only 5 of 13 lungs had recoverable CFU with the positive samples including two from group 2 (15.6 CFU/g and 1.51 x 10^7^ CFU/g), one from group 3 (25 CFU/g), and one from group 8 (2.27 x10^4^ CFU/g) (**Figure 6C**; **Supplementary Table 7**).

#### 3.3.4 Cell-mediated immune responses

##### 3.3.4.1 ELISpot assays

**Figure 7:**
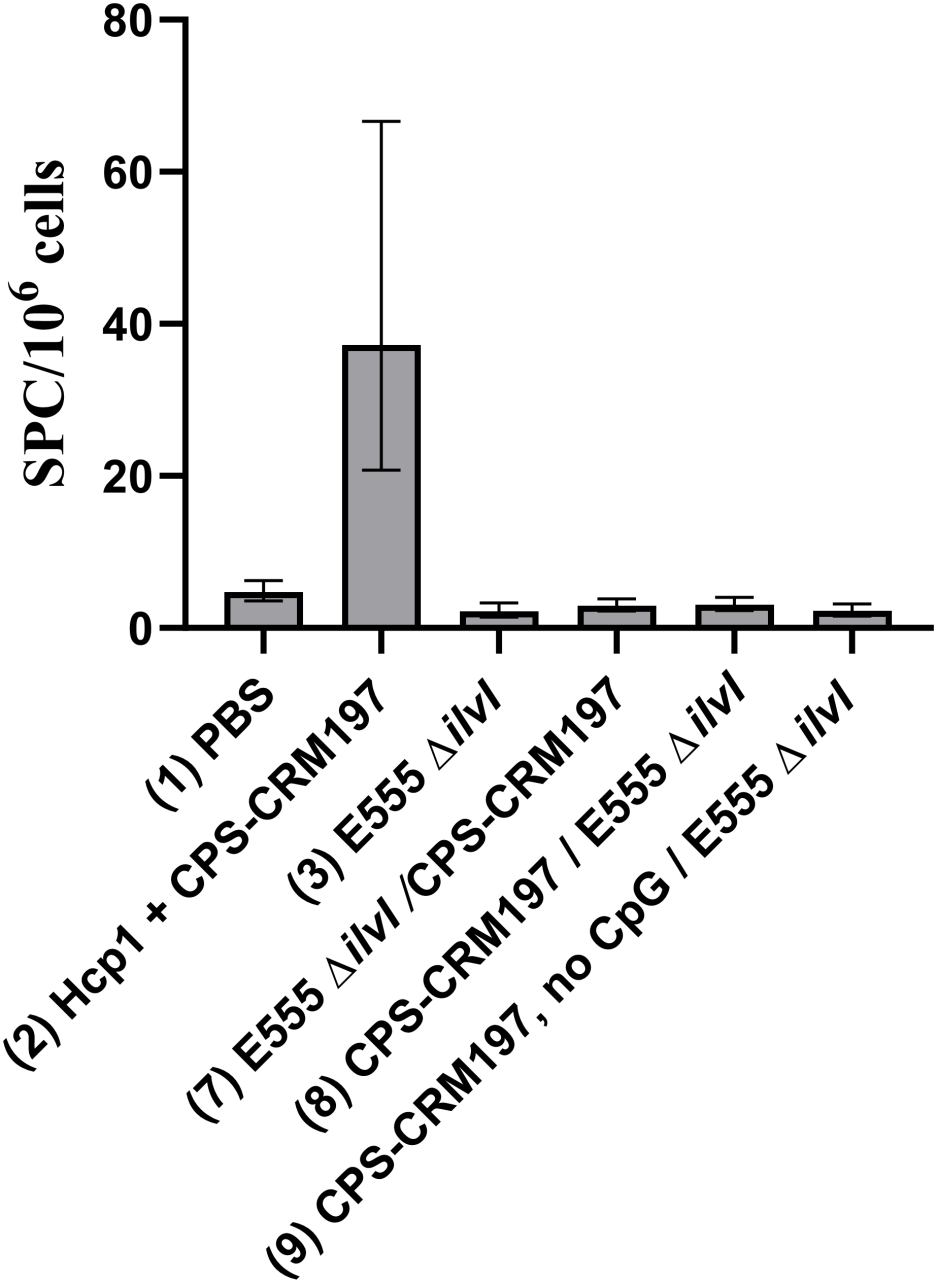
IFNγ-secreting splenocytes obtained from control and vaccinated C57BL/6 mice twenty-seven days post boost following stimulation with 10 µg/ml Hcp1. The splenocyte response of mice (*n* = 5/group) was assessed as spot forming cells (SFC), adjusted to 10^6^ cells per well. Values represent the GM ± the GSE.

Splenocytes isolated from spleens collected twenty-seven days after the boost dose were restimulated with Hcp1 or medium alone. Incubation of the cells in medium only yielded minimal numbers of IFN-γ-secreting cells, ranging from 1.22 - 2.99 SFC per 10^6^ cells (data not shown). As shown in **Figure 7**, the highest numbers of IFN-γ-secreting splenocytes after Hcp1 stimulation were produced by homologous vaccine group 2 (37.22 SFC/10^6^ cells, *p* < 0.0001 compared to the PBS controls), and the response indicated by elevated GM of group 2 was positively associated with protection (**Figure 5B**). The responses of the heterologous groups 7, 8, and 9, and homologous group 3 were not significantly different from that of the controls.

##### 3.3.4.2 Luminex assays

Luminex assays were used to measure cytokine production by splenocytes collected 27 days after the second vaccine dose. Assays of the cell supernatants were conducted after stimulation with 10 µg/ml Hcp1, and the cytokine results are shown as a ratio compared to the PBS controls (group 1). Following stimulation (**Supplementary Table 8**), group 2 exhibited a 1.5- to 2.3-fold increases in IL-2, IFN-γ, IL-17A, MCP-3 and IL-3 (*p* = 0.0124 for IFN-γ). Hcp1-stimulated splenocytes from groups 3, 7, and 8 exhibited down-regulated production of IL-2, MCP-3, and IP-10, most consistently in group 8 (*p* = 0.0005 to *p* = 0.0229) relative to the PBS controls. The vaccine groups also showed reduced levels of MIP-1α, MIP-1β, and RANTES in response to Hcp1 stimulation which was most consistently observed in group 8 (*p* < 0.0001 to *p* = 0.0116 for the three cytokines).

Three days following challenge, vaccines that conferred the greatest levels of protection (groups 2 and 8) also controlled cytokine induction to a greater extent relative to the three groups that were less efficacious (groups 3, 7, and 9), specifically for MIP-2α, IL-1β, MIP-1α, IL-1α, G-CSF, GRO-α, TNF-α, MIP-1β, IL-27, and MCP-3 (**Table 7**). These findings were suggestive of an attenuation of the cytokine storm by the more protective vaccine formulations.

**Table 7:** The cytokine responses in lung homogenates obtained three days after challenge with aerosolized *B. pseudomallei* K96243.

Cytokine^a^	Vaccine Group				
	(2) Hcp1 + CPS-CRM197	(3) E555 Δ*ilvI*	(7) E555 Δ*ilvI* / CPS-CRM197	(8) CPS-CRM197 / E555 Δ*ilvI*	(9) CPS-CRM197, no CpG / E555 Δ*ilvI*
IFNγ	*0.983*	*1.453*	*1.193*	*0.680*	**0.589**
RANTES (CCL5)	*0.967*	*1.063*	*0.994*	*1.033*	*1.018*
IL-31	*0.946*	*0.946*	*0.946*	*0.946*	*0.946*
IL-3	*0.860*	*0.860*	*0.875*	*0.908*	*0.860*
IL-12p70	*0.791*	**0.691**	**0.761**	**0.749**	**0.638**
IL-23	*0.710*	*0.774*	*0.887*	*0.958*	*0.866*
IL-28	*0.709*	**0.585**	*0.717*	*0.792*	**0.596**
IL-2	**0.704**	**0.729**	**0.796**	**0.736**	**0.728**
IL-15	**0.691**	**0.801**	*0.849*	**0.739**	**0.764**
IL-22	*0.660*	**0.127**	**0.167**	**0.223**	**0.112**
IL-9	**0.627**	**0.509**	**0.554**	**0.565**	**0.509**
IFN-α	**0.611**	**0.445**	**0.624**	**0.552**	**0.384**
Eotaxin	**0.593**	**0.557**	**0.593**	**0.622**	**0.596**
IL-4	**0.417**	**0.398**	**0.462**	**0.381**	**0.370**
IL-13	*0.400*	**0.345**	**0.482**	**0.311**	**0.394**
IP-10 (CXCL10)	**0.332**	**0.780**	**0.697**	**0.526**	**0.676**
IL-5	*0.253*	**0.123**	**0.322**	**0.163**	**0.155**
IL-10	**0.204**	**0.259**	**0.286**	**0.210**	**0.248**
MCP-3 (CCL7)	**0.198**	**0.533**	**0.477**	**0.327**	**0.444**
IL-27	**0.173**	**0.292**	**0.299**	**0.194**	**0.244**
ENA-78 (CXCL5)	**0.111**	**0.239**	**0.183**	**0.202**	**0.213**
MIP-1β (CCL4)	**0.106**	*0.270*	**0.264**	**0.119**	**0.308**
M-CSF	**0.097**	**0.128**	**0.176**	**0.090**	**0.187**
TNF-α	**0.090**	**0.169**	**0.190**	**0.097**	**0.165**
GRO-α (CXCL1)	**0.074**	**0.116**	**0.146**	**0.071**	**0.158**
IL-17A (CTLA-8)	**0.066**	**0.024**	**0.043**	**0.030**	**0.027**
LIF	**0.050**	**0.057**	**0.087**	**0.044**	**0.056**
G-CSF	**0.049**	**0.201**	**0.171**	**0.087**	**0.198**
IL-1α	**0.045**	**0.079**	**0.088**	**0.043**	**0.081**
GM-CSF	**0.030**	**0.041**	**0.052**	**0.031**	**0.037**
MIP-1α (CCL3)	**0.028**	**0.108**	**0.113**	**0.042**	**0.115**
IL-1β	**0.021**	**0.056**	**0.069**	**0.021**	**0.067**
MIP-2α (CXCL2)	**0.018**	**0.049**	**0.064**	**0.017**	**0.072**
IL-6	**0.009**	**0.011**	**0.018**	**0.009**	**0.013**

a The cytokine results are shown as the ratio to PBS, and are based on the Geometric Mean (pg/ml). Italicized and not bolded (not significant) and bolded (*p* < 0.05). The scale bar shows low responses in red and high responses in green. 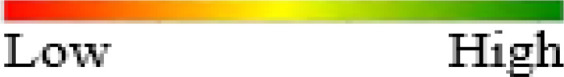

### 3.4 The protective efficacy of *B. pseudomallei* vaccines using pooled results to optimize the statistical analysis

**Figure 8:**
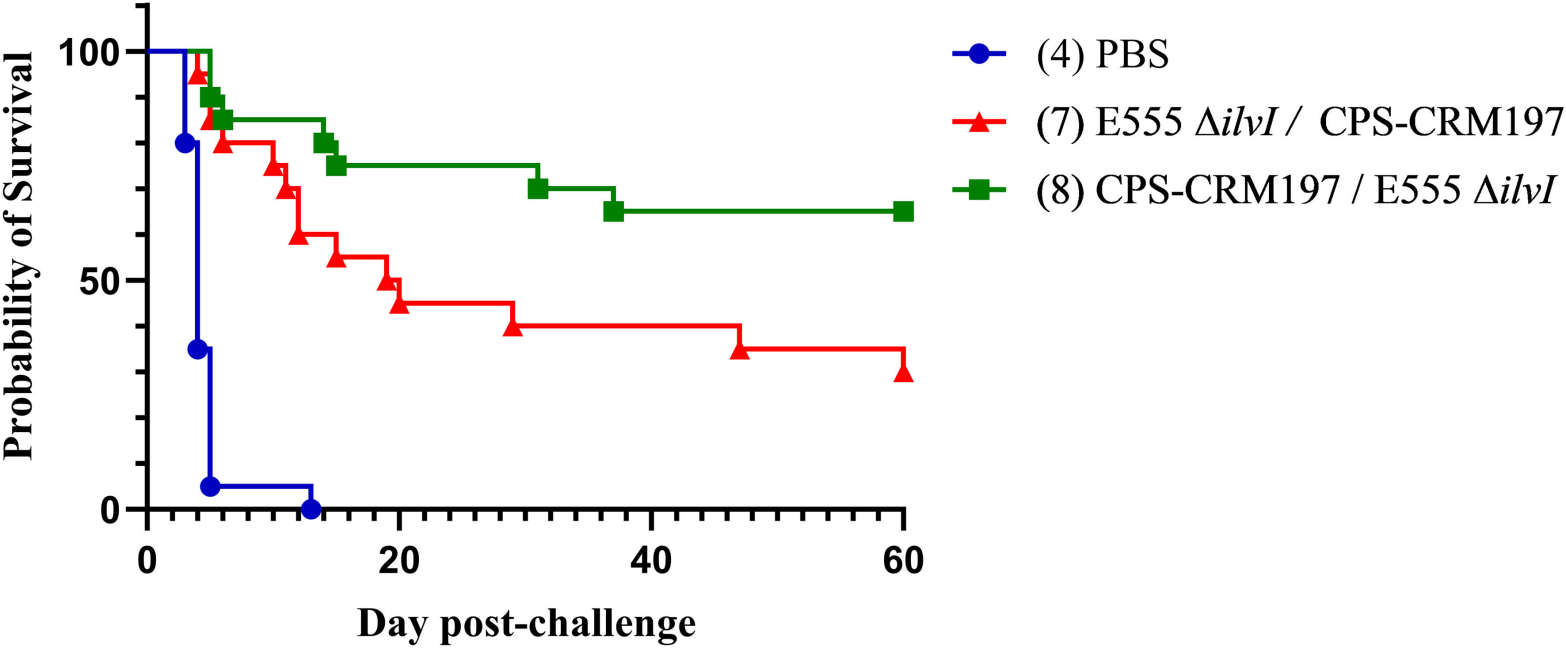
The combined survival data of mice from two experiments. The survival results of groups 7, 8, and the PBS controls, obtained from the studies depicted in **Figures 4**, **6**, are shown (*n* = 20/group). All mice had been challenged with the same whole body aerosol dose of *B. pseudomallei* K96243 (3.4 LD_50_s).

Since the challenge doses of *B. pseudomallei* K96243 used in the studies shown in **Figures 3C**, **5B** were identical, we pooled the data for vaccine group 8 (CPS-CRM197 followed by E555 Δ*ilvI*) and the PBS controls from these experiments for a more robust statistical analysis of protection. The reverse vaccination strategy group 7 (E555 Δ*ilvI* followed by CPS-CRM197) was included to assess the effects of dose order on efficacy. Both vaccination strategies were significantly protective through day 60 compared to the PBS controls (*p* = 0.0202 and *p* < 0.0001 for groups 7 and 8, respectively) and both vaccines significantly increased TTD as well (*p* < 0.0001). As shown in **Figure 8**, 13/20 mice (65%) receiving the group 8 vaccination strategy survived to day 60, whereas only 6/20 (30%) of the group 7 animals were protected and this difference approached significance (*p* = 0.056). In addition, the group 8 mice had a longer mean TTD compared to group 7 (*p* = 0.0353). Thus, the combined results for the heterologous strategy using a prime dose of CPS-CRM197 and boost dose of E555 Δ*ilvI* generated protection that appeared equal to that of the most protective homologous strategy (group 2) (**Figures 3B**, **5B**). Taken altogether, these data demonstrated that both homologous and heterologous strategies provided significant protection against a lethal inhalational challenge of *B. pseudomallei* in C57BL/6 mice. When all facets of the infection including survival kinetics, immunological responses, and bacterial clearance were considered the heterologous vaccination strategy appeared to be advantageous.

## 4 Discussion

### 4.1 Vaccine candidates for immunization against melioidosis

There are currently no licensed vaccines available for immunization against disease caused by *B. pseudomallei*, either in endemic settings or for biodefense purposes. Various pre-clinical studies have identified promising candidates that provide significant protection in mouse models of melioidosis. Prophylactic countermeasures have included defined subunit or inactivated whole cell vaccines as well as LAV strains. LAVs stimulate potentially broader immune responses in animal models and are often considered an optimal strategy for stimulating protection against *B. pseudomallei* infections (30, 32, 52–54). However, defined subunit vaccines are perceived to be safer and more amenable to large-scale production and such vaccines have provided significant protection in animal models (2, 33, 53, 55). Due to the complex facultative intracellular lifestyle of *B. pseudomallei*, a multi-component vaccine containing several bacterial antigens will likely be necessary to provide complete protection (4, 6, 32, 52, 56). To address the urgent need for melioidosis vaccines, our laboratories have developed both LAVs and defined subunit vaccines. In this study, we assessed 1) a LAV strain of *B. thailandensis* designated E555 Δ*ilvI* and 2) a subunit vaccine consisting of Hcp1 + CPS-CRM197, using homologous and heterologous immunization strategies.

*B. thailandensis* is a nonpathogenic, environmental species that is genetically related to *B. pseudomallei* (57, 58). *B. thailandensis* strain E555 produces a *B. pseudomallei*-like CPS (59, 60) and is considered to be a potential vaccine candidate for melioidosis since in past studies it stimulated high levels of anti-CPS antibodies and protected against a lethal challenge of *B. pseudomallei* in mice (57, 61). The E555 Δ*ilvI* LAV evaluated herein carries a deletion mutation which prevents synthesis of branched-chain amino acids to further augment its safety (30, 31). Several attenuated *B. pseudomallei* strains considered to be safe have been excluded from the select agent list (62). Examples of these included *B. pseudomallei* strains with mutations in *tonB* and *hcp1*, *asd*, *purM*, and a CPS biosynthetic gene (54, 63–67). We replaced our previously tested LAV strain *B. pseudomallei* 668 Δ*ilvI* (31) with the *B. thailandensis* E555 Δ*ilvI* mutant for increased safety and so that the LAV would not be considered biological select agent. The proof-of-concept evidence for the protective efficacy of *B. thailandensis* E555 Δ*ilvI* in BALB/c mice, as demonstrated by Klimko and coworkers (37), was confirmed in the current study with C57BL/6 mice. These results argue for renewed consideration of a LAV for melioidosis, either alone or in combination with a subunit(s) vaccine.

Previous studies have shown that a subunit vaccine consisting of the conserved 6-deoxyheptan CPS expressed by *B. pseudomallei* conjugated to carrier protein CRM197 and combined with recombinant Hcp1 (Hcp1 + CPS-CRM197) provided high level protection in mice that was comparable to that provided by LAV strains of *B. pseudomallei* (30–33). CPS is a major virulence factor and known protective antigen, and CPS-specific antibodies correlate with protection in mice (32, 34, 68, 69). Hcp1 is also a virulence factor and protective antigen in animal models as well as being a promising serodiagnostic target for detecting *B. pseudomallei* infections in humans (31, 45–48). The combination of CPS-CRM197 plus Hcp1 formulated with Alhydrogel and CpG was shown to be required for maximal efficacy of the vaccine against lethal inhalational *B. pseudomallei* challenges in mice (31, 33). The current investigation builds upon our previous research by 1) evaluating different combinations of the subunit vaccine antigens in parallel, 2) constructing and evaluating a potentially safer, effective *B. thailandensis* LAV, 3) determining the impact of vaccine order in a two-dose vaccination scheme, and 4) comparing the efficacy of homologous and heterologous vaccination schemes.

### 4.2 Comparison of protective efficacies and bacterial clearance-potential of the candidate vaccines

Comparisons of the overall protective efficacy of the current vaccines tested are summarized as follows: The *B. thailandensis* E555 Δ*ilvI* was shown here to be safe and stimulated comparable levels of protection to those previously reported for a *B. pseudomallei* 668 Δ*ilvI* LAV strain (31). It was efficacious alone (**Figures 1**, **2**) and in combination with a subunit vaccine (**Figures 3C**, **8**). The CPS-CRM197 vaccine was strongly protective, and the immunostimulant CpG maximized efficacy (as exemplified in **Figure 5B**), confirming prior findings (31, 70). The addition of Hcp1 to the subunit vaccine generally augmented protection in most cases (**Figures 1B**, **3C**, **5B**) (31, 70).

We also compared the efficacies of different vaccination strategies and the order of delivery of the various vaccine candidates. In the homologous strategies, both the Hcp1 + CPS-CRM197 vaccine and the LAV significantly enhanced survival compared to the PBS controls at the study endpoint (**Figure 3B**). Five different prime-boost combinations of heterologous vaccines were evaluated (**Table 3**), and all were significantly protective compared to the control mice when examining time to death or euthanasia and in some cases mortality rates. Furthermore, there were no detectable bacteria in the blood and significantly reduced bacterial loads in the lungs and spleens in vaccinated mice by 3 days post-challenge and were nearly or fully eradicated in the 60-day survivors (**Supplementary Table 3A**; **Figures 2**, **6**).

Protective efficacy was also impacted by the order of vaccine delivery. For example, when the CPS-CRM197 subunit vaccine (with immunostimulants) was administered first and E555 Δ*ilvI* given as a boost, protection was more than two-fold greater compared to the reverse order (**Figure 8**). The observation that vaccination with a subunit vaccine followed by a LAV is more protective has recently been described for heterologous vaccination strategies used to prevent pneumonic plague (71).

### 4.3 Comparison of Immune responses of the candidate vaccines

#### 4.3.1 Humoral immune responses

Overall, the E555 Δ*ilvI* vaccine stimulated the lowest levels of antibodies to BpK, CPS, and Hcp1 (**Tables 1**, **4A**, **5**). Masoud et al. reported that the majority of patients with different clinical manifestations of melioidosis had sera recognizing the CPS, which suggests that it is immunogenic and has the potential to be a vaccine or diagnostic antigen (68). Polysaccharides such as CPS are antigens that induce a T cell independent response and must be covalently attached to a carrier protein to elicit a strong adaptive immunity (32–34, 69). We hypothesize that the E555 Δ*ilvI* vaccine might not persist long enough in the host to generate a robust CPS IgG response; and perhaps a larger booster dose might be needed to prevent rapid clearance. Additionally, the native CPS being produced by the LAV will be less immunogenic when compared to purified CPS conjugated to the carrier CRM-197; this fact may also explain the disparate anti-CPS titers noted in vaccine strategies that contain the LAV strain as the sole antigenic source or when used as a booster to the subunit vaccine. Regarding anti-Hcp1 titers, the recombinant Hcp1 antigen used for the ELISA was purified from the cloned *B. pseudomallei* antigen (33). Although *B. thailandensis* and *B. pseudomallei* CPS are highly similar, the Hcp1 proteins from *B. thailandensis* and *B. pseudomallei* are not highly conserved at the amino acid level (46, 72, 73), possibly explaining the low anti-Hcp1 response in mice vaccinated with E555 Δ*ilvI*. In contrast, the subunit vaccines induced high titers to all antigens which were included in these vaccines (**Tables 1**, **4A**, **5**).

We also examined the immune response using irradiated *B. pseudomallei* K96243 (BpK) as the test antigen. The subunit vaccines elicited higher titers to BpK than did homologous LAV vaccines; these lower anti-BpK responses suggest the existence of important surface antigen differences between *B. pseudomallei* and *B. thailandensis* (74–76). The previously reported higher titers against BpK elicited by *B. pseudomallei* 668 Δ*ilvI* support this possibility (31).

Subclass analysis of IgG responses to the killed BpK antigen was performed. Whereas Hcp1 + CPS-CRM197 (homologous group 2) induced much higher anti-BpK IgG1 titers than the corresponding IgG2c levels, the reverse was observed for the homologous LAV (**Supplementary Table 6**; **Table 6**). These results confirmed our previous observations (31), in which CPS-CRM197 induced a Th2-skewed immune response whereas LAVs (*B. pseudomallei* 668 Δ*ilvI* and *B. thailandensis* E555 Δ*ilvI*) stimulated a more Th1-polarized response to whole cell inactivated *B. pseudomallei.*

The relative roles of the vaccine antigens, and of the specific immune responses to them, in protective efficacy is more difficult to dissect in the heterologously-vaccinated groups. All vaccines containing CPS-CRM197 in the prime or boost dose and CpG (groups 5 - 8), produced significant protection despite low antibody responses, including the most protective vaccination scheme, CPS-CRM197 prime followed by an E555 Δ*ilvI* boost (group 8, **Figures 3C**, **8**). The greater protection afforded by group 8 versus group 7 might be associated with their different anti-CPS responses (**Table 5**).

Nonetheless, humoral responses have an important role in murine survival and vaccine-mediated protection, as shown in active and passive transfer studies (33, 53, 69, 77–83). In our previous report, the high antibody titers to Hcp1 and CPS elicited by the subunit vaccine, but not the LAV, were protective (31). Finally, the recovery of humans with melioidosis has been associated with *B. pseudomallei*-specific humoral responses (84, 85); and Pumpuang et al. reported high levels of antibodies to Hcp1 in survivors (86). Since the pathogenesis of melioidosis involves both extra- and intra-cellular phases of *B. pseudomallei* infection, a logical prophylactic approach employs a combination of vaccine antigens which can stimulate both protective humoral and cell-mediated immune responses (31–33).

#### 4.3.2 Cellular immune responses.

Up-regulation of IFN-γ and downregulation of other pro-inflammatory cytokines appears to be important for protecting vaccinated animals against lethal *Burkholderia* infections. In the current study, the levels of IFN-γ in the lungs of mice were highest in those vaccinated with the LAV alone or combined with Hcp1 + CPS-CRM197 at three days after challenge (**Table 2**). All vaccine groups exhibited reduced levels of many pro-inflammatory cytokines in lungs compared to controls (such as TNF-α, IL-1β and IL-6), indicating that the vaccines prevented excessive expression of cytokines that can cause host damage, as described previously (31). The most efficacious vaccine formulations were those containing CPS-CRM197 in the prime dose (groups 2 and 8) which resulted in more controlled levels of cytokine expression than did the less protective vaccines (groups 3, 7, and 9) (**Table 7**). The cytokine and chemokine responses in sera, lungs, and reticuloendothelial organs of naïve mice infected with *B. pseudomallei* have been extensively characterized, as summarized recently (87). Although laboratory-associated differences in timing and specificity of cytokine production were apparent, most described a rapid increase of proinflammatory cytokine levels within one to three days in lungs, spleen, and sera, with a subsequent decline (40, 41, 88–91).

IFN-γ is a major Th1 cytokine involved in stimulating proinflammatory cytokines, and macrophage phagocytic activity is a likely correlate of survival in mouse models of melioidosis (30, 31, 33, 53, 92–95). The association of protection with strong IFN-γ responses in vaccinated mice is consistent with observations in humans with melioidosis (96, 97). Specifically, the secretion of IFN-γ by CD4+ T cells from melioidosis patients, when stimulated with Hcp1 and TssM, was associated with improved survival (98, 99). In contrast, elevated levels of anti-Hcp1 IgG were not associated with patient survival (98) which supports a role for IFN-γ and T cell immunity in recovery from melioidosis (32, 98, 100).

## 5 Conclusions

Taken together, the data from the current study demonstrates that both homologous and heterologous vaccination strategies provided significant protection against a lethal inhalational challenge of *B. pseudomallei* in mice. The heterologous vaccination approach may, however, be beneficial when considering multiple facets of the infection including survival kinetics, immunological responses, and bacterial clearance. Overall, the data obtained in this study confirm previous findings (31, 33) and support that the heterologous vaccination strategy using Hcp1 + CPS-CRM197 (with Alhydrogel plus CpG) and the LAV strain may be optimal. Future work will involve vaccine optimization to include examination of administration routes that could increase mucosal immune responses. Lastly, we acknowledge the inherent difficulty associated with heterologous vaccination strategies. These concerns can range from vaccine characterization, preparation, administration, and safety concerns. At the same time, these proof-of-concept data can be leveraged to generate novel, agile vaccination strategies to prevent emerging or re-emerging bacterial diseases important to both the biodefense community and the public health arena, such as melioidosis.

## Data Availability

The raw data supporting the conclusions of this article will be made available by the authors, without undue reservation.
